# DNA single-strand break-induced DNA damage response causes heart failure

**DOI:** 10.1038/ncomms15104

**Published:** 2017-04-24

**Authors:** Tomoaki Higo, Atsuhiko T. Naito, Tomokazu Sumida, Masato Shibamoto, Katsuki Okada, Seitaro Nomura, Akito Nakagawa, Toshihiro Yamaguchi, Taku Sakai, Akihito Hashimoto, Yuki Kuramoto, Masamichi Ito, Shungo Hikoso, Hiroshi Akazawa, Jong-Kook Lee, Ichiro Shiojima, Peter J. McKinnon, Yasushi Sakata, Issei Komuro

**Affiliations:** 1 Department of Cardiovascular Medicine, Osaka University Graduate School of Medicine, 2-2 Yamadaoka, Suita 565-0871, Japan; 2 CREST, Sanbanmachi-building, 5 Sanbanmachi, Tokyo 102-0075, Japan; 3 Department of Cardiovascular Medicine, The University of Tokyo Graduate School of Medicine, 7-3-1 Hongo, Tokyo 113-8655, Japan; 4 Department of Medicine II, Kansai Medical University, 2-5-1 Shinmachi, Hirakata 573-1191, Japan; 5 Department of Genetics and Tumor Cell Biology, ST. Jude Children's Research Hospital, 262 Danny Thomas Place, Memphis, Tennessee 38105, USA; 6 Institute for Academic Initiatives, Osaka University, 2-2 Yamadaoka, Suita 565-0871, Japan

## Abstract

The DNA damage response (DDR) plays a pivotal role in maintaining genome integrity. DNA damage and DDR activation are observed in the failing heart, however, the type of DNA damage and its role in the pathogenesis of heart failure remain elusive. Here we show the critical role of DNA single-strand break (SSB) in the pathogenesis of pressure overload-induced heart failure. Accumulation of unrepaired SSB is observed in cardiomyocytes of the failing heart. Unrepaired SSB activates DDR and increases the expression of inflammatory cytokines through NF-κB signalling. Pressure overload-induced heart failure is more severe in the mice lacking XRCC1, an essential protein for SSB repair, which is rescued by blocking DDR activation through genetic deletion of ATM, suggesting the causative role of SSB accumulation and DDR activation in the pathogenesis of heart failure. Prevention of SSB accumulation or persistent DDR activation may become a new therapeutic strategy against heart failure.

## Abstract (web summary)

DNA damage response (DDR) is activated in cardiomyocytes of the failing heart, but the type of DNA damage leading to DDR is unclear. Higo *et al*. show that in mice heart failure is caused in part by unrepaired DNA single-strand breaks in cardiomyocytes, which activate persistent DDR and trigger an NF-κB-dependent cardiac inflammation.

Various internal and external stresses induce many types of DNA damage such as chemical change of bases, single-strand break (SSB) and double-strand break (DSB). On DNA damage, the DNA damage response (DDR) signalling is activated to repair damaged DNA. Recruitment and autophosphorylation of ataxia telangiectasia mutated (ATM) kinase are one of the most well characterized DDR, which are usually activated after DNA DSB^1^. Phosphorylated ATM in turn phosphorylates multiple effector proteins including the histone variant H2AX and tumor suppressor p53, and triggers downstream signalling pathways that stop the cell cycle and repair damaged DNA^2^,^3^. When the DNA damage is too extensive to be repaired, cells usually undergo apoptosis or stop the cell cycle permanently, which is termed cellular senescence^2^,^4^. Activation of DDR is observed not only in mitotic cells but also in post-mitotic cells including cardiomyocytes^5^,^6^,^7^. Various types of DNA damage including DNA oxidation, SSB and DSB are observed in the infarcted heart and activation of DDR plays an important role in cardiac remodelling after myocardial infarction through inducing cardiomyocyte apoptosis^7^,^8^,^9^. Activation of DDR is also observed in cardiomyocytes of the patients with end-stage heart failure^5^ and the pressure-overload induced heart failure model mice^6^, however, the type of DNA damage and the role of DDR in the pathophysiology of heart failure remain unclear.

In the present study, we show that DNA SSB, but not DNA DSB, is accumulated in cardiomyocytes of pressure-overload induced heart failure model mice. Accumulation of DNA SSB activates persistent DDR and induces inflammatory gene expression in an NF-κB-dependent manner. Cardiomyocyte-specific gene deletion of *Xrcc1*, an essential protein for SSB repair, leads to more severe cardiac inflammation and heart failure after pressure-overload, which is rescued by simultaneous deletion of *Atm* gene. These results suggest the unrecognized role of DNA SSB in the pathogenesis of pressure overload-induced heart failure.

## Results

### DNA single-strand break accumulation in heart failure

**Figure 1:**
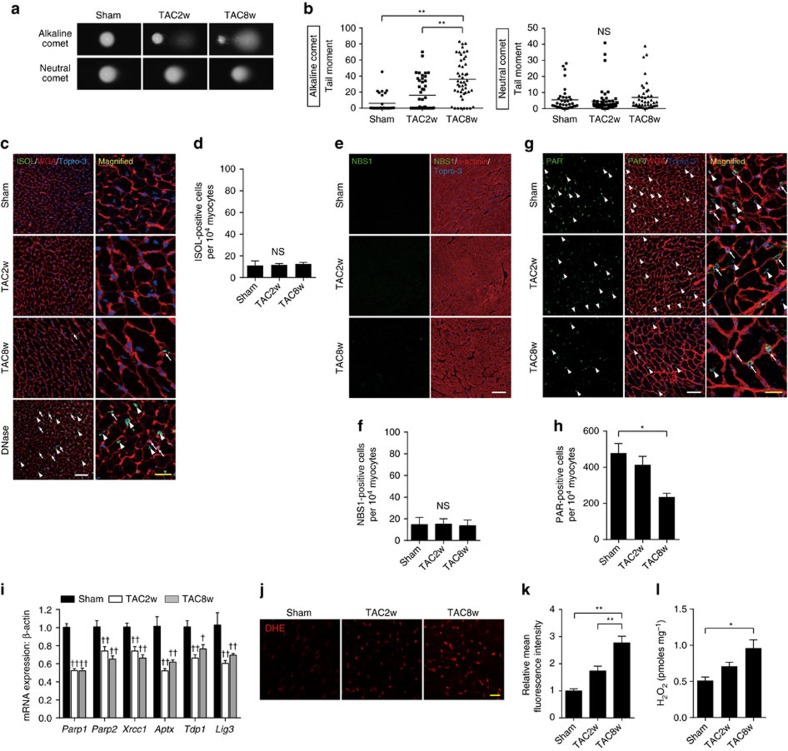
Accumulation of DNA SSB in the failing heart. (**a**,**b**) Cardiomyocytes were isolated from the TAC-operated heart at the indicated time points. The type of DNA damage in cardiomyocytes was assessed by comet assay. Representative images (**a**) and quantitative analyses are shown (**b**, Alkaline comet: *n*=28, 45, 48; Neutral comet: *n*=38, 56, 44 at each time point, respectively, biological replicates=3). (**c**,**d**) Fragmented DNA and DSB were labelled with ISOL staining (**c**, green). Wheat germ agglutinin (WGA, red) was used to visualize cardiomyocytes. DNase-treated section (DNase I, 10 Kunitz units ml^−1^) was used as a positive control. Arrowheads indicate ISOL-positive cardiomyocytes and arrows indicate ISOL-positive non-cardiomyocytes. White scale bar, 50 μm; yellow scale bar, 20 μm. The number of ISOL-positive cardiomyocytes was counted (**d**, *n*=4 each). (**e**,**f**) Heart tissue sections were immunostained for NBS1 (**e**, NBS1, green). Immunostaining for alpha-actinin (red) was used to label cardiomyocytes. Scale bar, 50 μm. The number of NBS1-positive cardiomyocytes was counted (**f**, *n*=4 each). (**g**,**h**) Heart tissue sections were immunostained for poly-ADP ribose (**g**, PAR, green) and the number of PAR-positive cardiomyocytes was counted (**h**, *n*=4, 4, 5 at each time point, respectively). Arrowheads indicate PAR-positive cardiomyocytes and arrows indicate PAR-positive non-cardiomyocytes. White scale bar, 50 μm; yellow scale bar, 20 μm. (**i**) Expression levels of SSB repair enzymes were analysed by real-time PCR (*n*=4, 6, 8 at each time point, respectively, technical duplicates). (**j**,**k**) Heart tissue sections were stained with dihydroethidium (**i**, DHE, 10 μΜ) and mean fluorescence intensity relative to Sham-operated mice was measured (**k**, *n*=4, 5, 5 at each time point, respectively). Scale bar, 50 μm. (**l**) The level of H_2_O_2_ in the TAC-operated heart was measured using Amplex Red assay (*n*=9, 5, 6 at each time point, respectively). Statistical significance was determined by Steel-Dwass test for (**b**) and by one-way analysis of variance followed by the Tukey–Kramer HSD test for (**d**,**f**,**h**,**i**,**j**) *P<0.05; **P<0.01 between arbitrary two groups. ^†^*P*<0.05; ^††^*P*<0.01 versus Sham. Column and error bars show mean and s.e.m., respectively.

We performed transverse aortic constriction (TAC) surgery on mice to induce pressure overload-induced heart failure. Left ventricular hypertrophy was observed in 2 weeks, and progressive cardiac dysfunction was observed in 8–10 weeks after the TAC surgery (Supplementary Fig. 1). To elucidate the type of DNA damage in the failing heart, we isolated cardiomyocytes after the TAC surgery and analysed the type of DNA damage by comet assay. Comet tail moment in the alkaline condition (alkaline comet) reflects both DNA DSB and SSB whereas comet tail moment in the neutral condition (neutral comet) reflects only DNA DSB^10^. To optimize the experimental condition of the comet assays, we used cardiomyocytes isolated from doxorubicin-treated mice and confirmed that neutral comet assay could indeed detect DNA DSB in the heart (Supplementary Fig. 2a). We found that the alkaline comet tail moment of cardiomyocytes was increased time dependently after TAC operation whereas neutral comet tail moment was comparable between Sham- and TAC-operated mice (Fig. 1a,b), suggesting that the number of cardiomyocytes with more SSB, but not DSB, is increased in the heart after pressure overload. To further analyse the type of DNA damage in the failing heart, we performed *in situ* oligo ligation (ISOL), which enables in situ detection of DNA DSB^11^, and immunofluorescent staining for NBS1, which is a component of MRE11-RAD50-NBS1 DSB repair complex and used as a DNA DSB marker^12^. The number of ISOL- and NBS1-positive cardiomyocytes was increased in the heart after myocardial infarction (Supplementary Fig. 2b,c) whereas they were not increased in the heart after TAC operation (Fig. 1c–f), underscoring the results of the comet assay. Notably, the number of ISOL-positive non-cardiomyocytes was increased after TAC operation (Supplementary Fig. 2d). We cannot totally exclude the possibility that the cells with DNA DSB are cleared by phagocytes too quickly to be detected by these assays (neutral comet assay, ISOL and NBS1 staining), or the sensitivity of these assays were not enough to detect DNA DSB in cardiomyocytes, however, it is very likely that the major type of DNA damage that accumulates in cardiomyocytes after pressure-overload is DNA SSB, but not DSB.

Despite our observation that DNA SSB was increased in cardiomyocytes after TAC operation, we found that the level of protein poly(ADP-ribosylation), which is induced by PARP1/2 as an initial process of SSB repair^13^, and the expression levels of SSB repair-related genes were rather decreased in the TAC-operated heart (Fig. 1g-i). Together with the findings that the levels of reactive oxygen species (ROS) were significantly increased (Fig. 1j–l), decreased SSB repair activity as well as increased ROS production may play a role in SSB accumulation in the heart after TAC operation (Supplementary Fig. 3).

### Xrcc1 deficiency exacerbates heart failure

**Figure 2:**
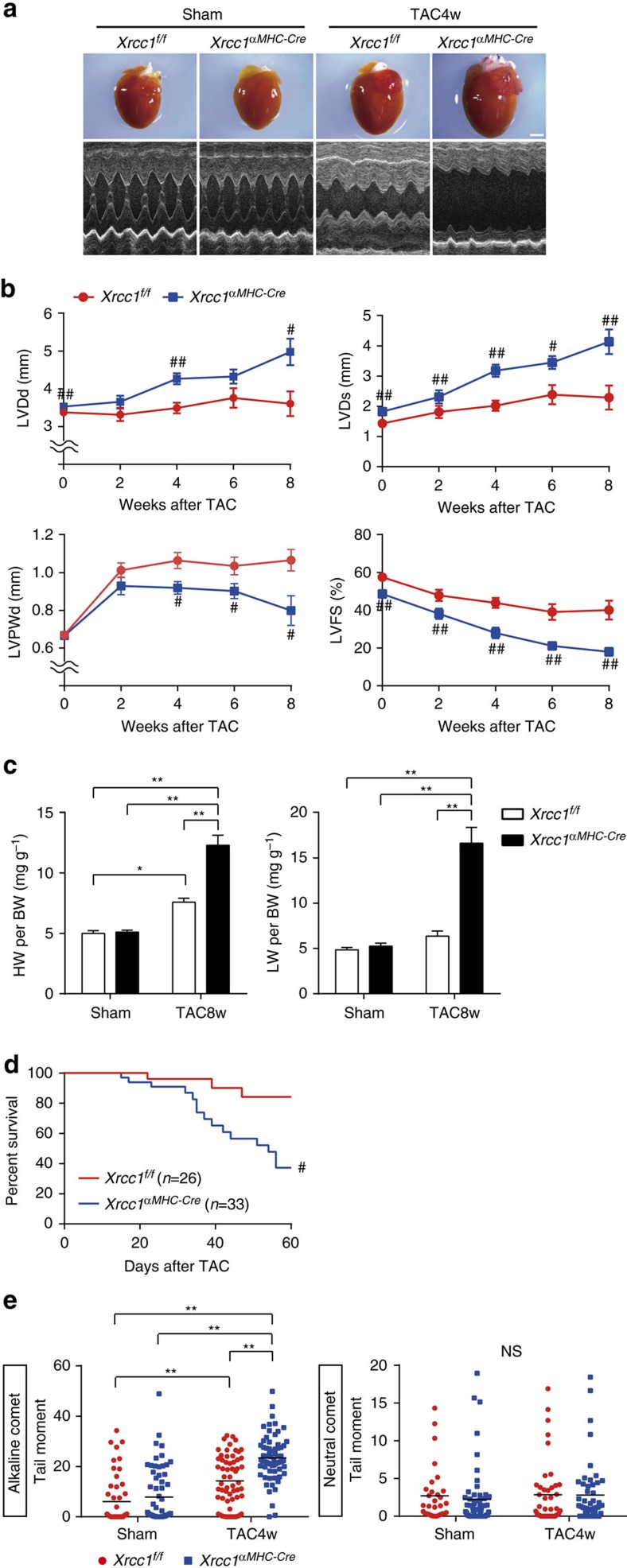
Xrcc1 deficiency increase SSB accumulation and exacerbates heart failure. (**a**) Macroscopic and echocardiographic images of Sham- or TAC-operated *Xrcc1*^*f/f*^ and *Xrcc1*^*αMHC-Cre*^ mice. Scale bar, 2 mm. (**b**) TAC surgery was performed to *Xrcc1*^*f/f*^ and *Xrcc1*^*αMHC-Cre*^ mice and cardiac function after the operation was assessed by echocardiogram. LVDd, LV end-diastolic dimension; LVDs, LV end-systolic dimension; LVPWd, LV posterior wall dimension; LVFS, LV fractional shortening (*Xrcc1*^*f/f*^ mice: *n*=80, 22, 30, 11, 10; *Xrcc1*^*αMHC-Cre*^ mice: *n*=85, 28, 40, 13, 9 at each time point, respectively). Statistical significance was determined by Student's *t*-test at each time point. ^#^*P*<0.05; ^##^*P*<0.01 versus *Xrcc1*^*f/f*^ mice. (**c**) Heart, lung, and body weight of Sham- or TAC-operated *Xrcc1*^*f/f*^ and *Xrcc1*^*αMHC-Cre*^ mice were weighed 8 weeks after the TAC surgery (*n*=8, 9, 12, 7, respectively). Statistical significance was determined by one-way analysis of variance followed by the Tukey–Kramer HSD test. *P<0.05; **P<0.01 between arbitrary two groups. (**d**) Survival curve of *Xrcc1*^*f/f*^ and *Xrcc1*^*αMHC-Cre*^ mice after the TAC surgery (*n*=26, 33, respectively). Statistical significance was determined by Wilcoxon test. ^#^*P*<0.05 versus *Xrcc1*^*f/f*^ mice. (**e**) The type of DNA damage in cardiomyocytes of Sham- or TAC-operated *Xrcc1*^*f/f*^ and *Xrcc1*^*αMHC-Cre*^ mice was assessed by comet assay (Alkaline comet: *n*=50, 64, 60, 67; Neutral comet: *n*=31, 57, 42, 50, respectively). Statistical significance was determined by Steel–Dwass test. **P<0.01 between arbitrary two groups. Column and error bars show mean and s.e.m., respectively.

In mitotic cells, unrepaired SSB usually does not accumulate but develops into DNA DSB and induces catastrophic cell death during mitotic phase^14^. Therefore, accumulation of SSB is supposed to be a unique phenomenon observed only in post-mitotic cells^15^. To examine the causal relation between SSB accumulation and heart failure, we generated the mice with defective SSB repair. X-ray repair complementing defective repair in Chinese hamster cells 1 (XRCC1) is a scaffold protein that interacts with various SSB repair enzymes and essential for SSB repair^16^. Since *Xrcc1* knockout mice exhibit early embryonic lethality due to massive DNA damage and cellular apoptosis^17^, we crossed the mice homozygous for an *Xrcc1*^*flox*^ allele with transgenic mice expressing *Cre*-recombinase under the control of the α-myosin heavy chain promoter (α-myosin *MHC-Cre*^*tg*^)^18^,^19^ and obtained *Xrcc1*^*flox/flox*^; α*MHC-Cre*^*tg*^ (*Xrcc1*^*αMHC-Cre*^) mice as cardiomyocyte-specific *Xrcc1* knockout mice. *Xrcc1*^*flox/flox*^; α*MHC-Cre*^*−*^ mice (*Xrcc1*^*f/f*^) were used as controls. Deletion of *Xrcc1* in the heart tissue of *Xrcc1*^*αMHC-Cre*^ mice was confirmed at the DNA, mRNA and protein levels (Supplementary Fig. 4a–c). *Xrcc1*^*αMHC-Cre*^ mice were born in expected Mendelian ratios, and were viable and fertile. Echocardiographic analysis revealed mild cardiac dysfunction in *Xrcc1*^*αMHC-Cre*^mice (Supplementary Fig. 4d), but the mice showed no overt signs of heart failure such as tachypnea, weight loss and pleural effusions beyond 1-year follow-up (Supplementary Fig. 4e), suggesting that deletion of *Xrcc1* in cardiomyocytes is not sufficient to induce heart failure. After the TAC surgery, however, *Xrcc1*^*αMHC-Cre*^ mice exhibited more severe left ventricular dilatation and dysfunction (Fig. 2a,b), more severe signs of heart failure (Fig. 2c) and higher mortality (Fig. 2d) compared with *Xrcc1*^*f/f*^ mice.

Comet assay revealed that the level of SSB in cardiomyocytes tended to increase, but statistically comparable between sham-operated *Xrcc1*^*αMHC-Cre*^ and *Xrcc1*^*f/f*^ mice (Fig. 2e), suggesting that cardiomyocyte-specific deletion of *Xrcc1* by itself is not sufficient to induce massive SSB accumulation in the non-stressed heart. After the TAC operation, however, the level of SSB in cardiomyocytes was more increased in the heart of *Xrcc1*^*αMHC-Cre*^ mice compared with *Xrcc1*^*f/f*^ mice (Fig. 2e), whereas the level of DSB in cardiomyocytes was comparable between *Xrcc1*^*αMHC-Cre*^ and *Xrcc1*^*f/f*^ mice (Supplementary Fig. 5). These results collectively suggest that insufficient SSB repair due to deletion of *Xrcc1* induces more SSB accumulation and exacerbates cardiac dysfunction in response to pressure overload and also indicate that SSB accumulation is tightly associated with the progression of pressure overload-induced heart failure.

### SSB activates DDR and induce inflammation through NF-κB

**Figure 3:**
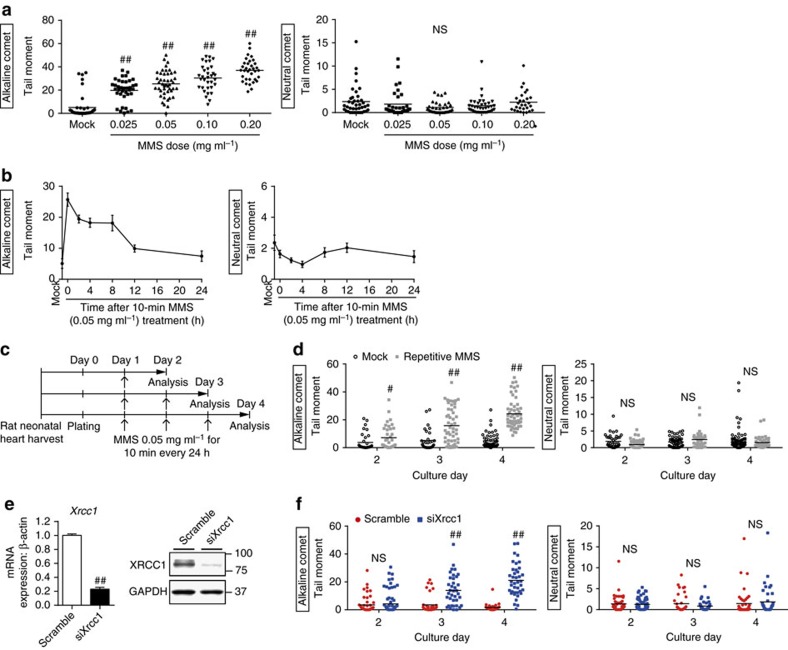
Generation of an *in vitro* model of cardiomyocytes with SSB accumulation. (**a**) Neonatal rat cardiomyocytes (NRCMs) were treated with MMS at the indicated concentration for 10 min and the DNA damage was analysed by comet assay (Alkaline comet: *n*=42, 37, 45, 33, 34; Neutral comet: *n*=40, 35, 35, 37, 29 at each concentration, respectively). Statistical significance was determined by Steel–Dwass test. ^##^*P*<0.01 versus Mock. (**b**) NRCMs were treated with MMS (0.05 mg ml^−1^ for 10 min) and the DNA damage was analysed by comet assay at the indicated time point (Alkaline comet: *n*=41, 21, 31, 29, 16, 81, 30; Neutral comet: *n*=40, 36, 41, 34, 35, 41, 56 at each time point, respectively). (**c**) Schedule for repetitive MMS treatment. (**d**) NRCMs were subjected to repetitive MMS treatment as described in **c** and the DNA damage was analysed by comet assay (Alkaline comet: *n*=35, 37, 32, 49, 52, 54; Neutral comet: *n*=47, 54, 55, 38, 68, 45 at each time point, respectively). Statistical significance was determined by Mann-Whitney *U* test. ^#^*P*<0.05 and ^##^*P*<0.01 versus Mock at each time point. (**e**,**f**) NRCMs were transfected with siRNA against *Xrcc1* (siXrcc1) or scrambled oligonucleotide (Scramble) as a control. Knockdown efficiency of siRNA against *Xrcc1* was examined by real-time PCR and western blotting (**e**, *n*=6, 8, technical duplicates). Time-dependent changes of DNA damage after the knockdown of *Xrcc1* was assessed by comet assay (**f**, Alkaline comet: *n*=52, 76, 38, 37, 34, 39; Neutral comet: *n*=52, 65, 37, 36, 45, 41 at each time point, respectively). Statistical significance was determined by Mann–Whitney *U*-test for (**e**,**f**) ^##^*P*<0.01 versus Scramble at each time point. Column and error bars show mean and s.e.m., respectively.

To elucidate the molecular mechanism by which SSB accumulation in cardiomyocytes exacerbates pressure overload-induced heart failure, we established an *in vitro* model of cardiomyocytes with SSB accumulation by using alkylating agent methyl methanesulfonate (MMS), which induces only DNA SSB^20^, and small interfering RNA (siRNA)-mediated knockdown of *Xrcc1*. To optimize the experimental condition of the comet assays and ISOL staining in *in vitro* cultured cardiomyocytes, we used irradiation to induce DNA DSB and confirmed that DNA DSB can be detected in both assays (Supplementary Fig. 6a,b). We observed concentration-dependent, temporal SSB after 10-min treatment with MMS (Fig. 3a,b) and repetitive treatment with MMS generated unrepaired SSB accumulation in cultured cardiomyocytes (Fig. 3c,d). Knockdown of *Xrcc1* (Fig. 3e) also generated a time-dependent accumulation of SSB in cultured cardiomyocytes (Fig. 3f). DNA DSB was not induced either by MMS treatment or by knockdown of *Xrcc1* (Fig. 3a,b,d,f and Supplementary Fig. 6a–d).

**Figure 4:**
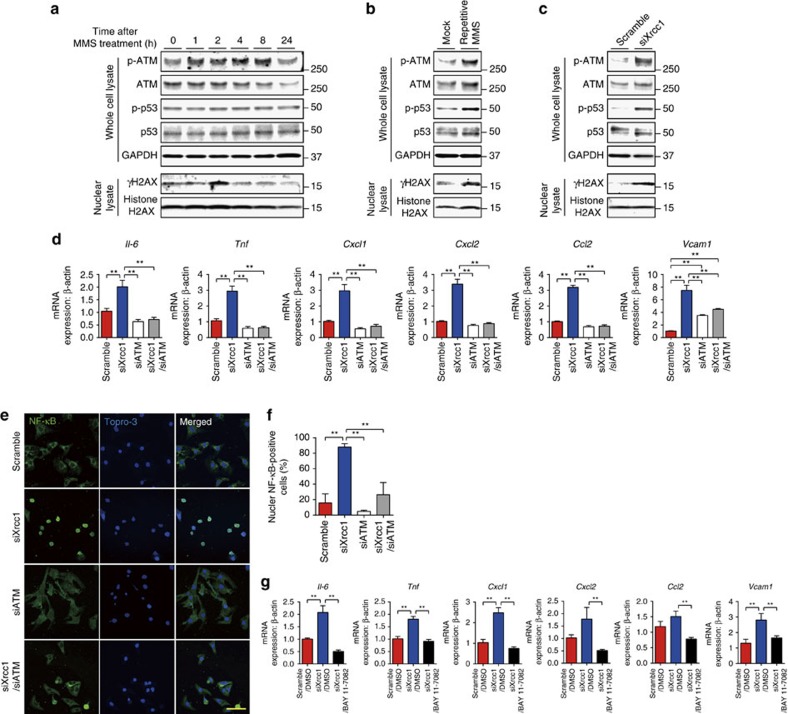
SSB activates DDR and induce inflammation through NF-κB. (**a**) Neonatal rat cardiomyocytes (NRCMs) were treated with MMS (0.05 mg ml^−1^ for 10 min) or vehicle control (Mock) and activation of DDR was assessed by western blotting against phospho- or total ATM, H2AX and p53 at the indicated time point. Western blotting against GAPDH was used as a loading control. (**b**) NRCMs were treated with MMS (0.05 mg ml^−1^ for 10 min) or vehicle alone (Mock) for 3 consecutive days and activation of DDR was assessed as described in **a**. (**c**) NRCMs were transfected with siRNA against *Xrcc1* (siXrcc1) or scrambled negative control oligonucleotide (Scramble). Four days later, activation of DDR was assessed as described in **a**. (**d–f**) NRCMs were transfected with siRNA against *Xrcc1* and/or *Atm.* Expression levels of inflammatory cytokines were assessed by real-time PCR (**d**, *n*=6 each, technical duplicates). Nuclear translocation of NF-κB was assessed by immunofluorescence (**e**, green). The nuclei of the cells were counterstained with TO-PRO-3 iodide 642/661 (blue). Scale bar, 20 μm. Cells with positive nuclear NF-κB staining were counted (**f**, *n*=7, 8, 8, 5, respectively). Statistical significance was determined by one-way analysis of variance (ANOVA) followed by the Tukey-Kramer HSD test for (**d**,**f**) **P<0.01 between arbitrary two groups. (**g**) NRCMs were transfected with siRNA against *Xrcc1* and treated with NF-κB inhibitor BAY 11-7082 (2 μM) or dimethylsulfoxide as a vehicle control. The expression levels of inflammatory cytokines were analysed by real-time PCR (*n*=6 each, technical duplicates). Statistical significance was determined by one-way ANOVA followed by the Tukey–Kramer HSD test. **P<0.01 between arbitrary two groups. Column and error bars show mean and s.e.m., respectively.

We then analysed how DDR is activated after SSB induction. Single treatment with MMS induced phosphorylation of ATM and H2AX whereas phosphorylation of p53 was not observed (Fig. 4a). Repetitive treatment with MMS or knockdown of *Xrcc1*, however, induced phosphorylation of p53 as well as ATM and H2AX (Fig. 4b,c) that resembles to the DDR after ionizing radiation or doxorubicin, which causes DNA DSB (Supplementary Fig. 7a,b). Persistent activation of DDR usually induces apoptotic cell death or cellular senescence^21^. Recent reports suggest that senescent cells show inflammatory phenotype, which is termed senescence-associated secretory phenotype (SASP)^22^,^23^. Therefore, we tested whether SSB accumulation and persistent activation of DDR induce inflammatory phenotype in cardiomyocytes. Knockdown of *Xrcc1* increased the expression of inflammatory cytokines such as *Il6, Cxcl1, Ccl2* and *Vcam1* in cardiomyocytes suggesting that SSB accumulation leads to acquisition of inflammatory phenotype in cardiomyocytes. Persistent phosphorylation/activation of ATM plays a central role in acquisition of SASP in senescent cells^4^. Persistent activation of DDR and increased expression of inflammatory cytokines after knockdown of *Xrcc1* were abolished by simultaneous knockdown of *Atm* (Fig. 4d and Supplementary Fig. 7c), suggesting that ATM is essential for SSB accumulation-induced acquisition of inflammatory phenotype in cardiomyocytes.

NF-κB plays a key role during inflammation. Previous report also suggests the involvement of NF-κB in DDR^24^,^25^,^26^. We also observed that the number of cardiomyocytes with nuclear NF-κB staining was increased after knockdown of *Xrcc1* and was abolished by simultaneous knockdown of *Atm* (Fig. 4e,f). BAY 11-7082, an inhibitor of NF-κB, blocked SSB accumulation-induced NF-κB activation and inflammatory gene expression in cardiomyocytes (Fig. 4g and Supplementary Fig. 7d,e). These results collectively suggest that SSB accumulation induces inflammatory cytokine expression through persistent activation of DDR and subsequent activation of NF-κB pathway in cardiomyocytes.

### Xrcc1 deficiency exacerbates DDR and cardiac inflammation

**Figure 5:**
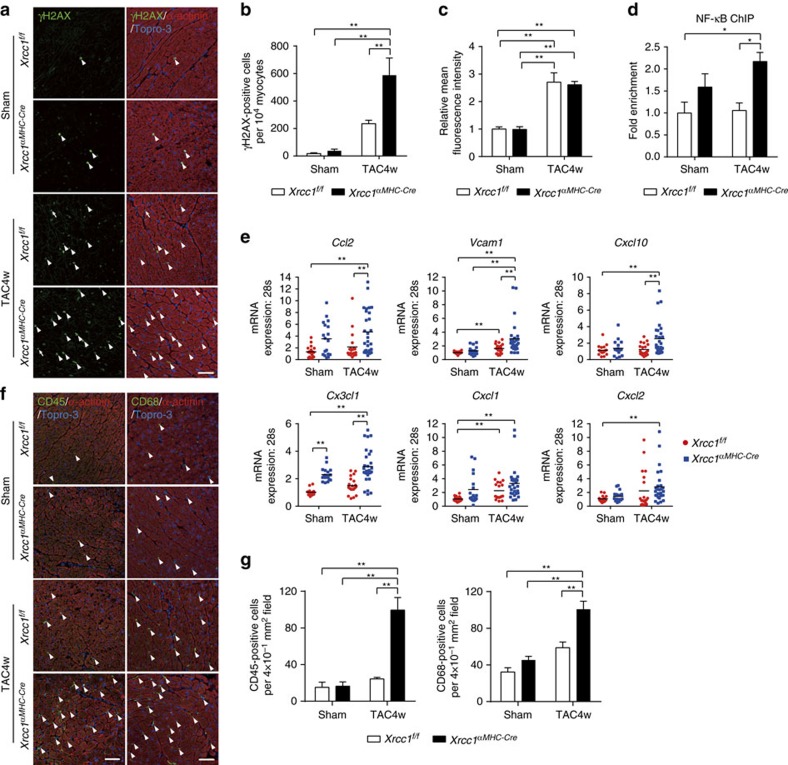
*Xrcc1* deficiency exacerbates cardiac inflammation after pressure overload. (**a**,**b**) Activation of DDR in Sham- or TAC-operated *Xrcc1*^*f/f*^ and *Xrcc1*^*αMHC-Cre*^ mice was assessed by immunostaining for phosphorylated H2AX (**a**, γH2AX, green). Immunostaining for alpha-actinin (red) was used to label cardiomyocytes. Arrowheads indicate γH2AX-positive cardiomyocytes and arrows indicate γH2AX-positive non-cardiomyocytes. Scale bar, 50 μm. The number of γH2AX-positive cardiomyocytes was counted (**b**, *n*=4 each). Statistical significance was determined by one-way analysis of variance (ANOVA) followed by the Tukey–Kramer HSD test. **P<0.01 between arbitrary two groups. (**c**) Heart tissue sections were stained with dihydroethidium and mean fluorescence intensity relative to Sham-operated *Xrcc1*^*f/f*^ mice was measured (*n*=5 each). Statistical significance was determined by one-way ANOVA followed by the Tukey–Kramer HSD test. **P<0.01 between arbitrary two groups. (**d**) ChIP–qPCR analysis of binding of NF-κB to the *Vcam1* promoter region in Sham- or TAC-operated *Xrcc1*^*f/f*^ and *Xrcc1*^*αMHC-Cre*^ mice. Data are presented as fold enrichment relative to Sham-operated *Xrcc1*^*f/f*^ mice (*n*=4, 4, 5, 5, respectively). Statistical significance was determined by one-way ANOVA followed by the Tukey–Kramer HSD test. *P<0.05 between arbitrary two groups. (**e**) The expression levels of inflammatory cytokines in the isolated cardiomyocytes of Sham- or TAC-operated *Xrcc1*^*f/f*^ and *Xrcc1*^*αMHC-Cre*^ mice was assessed by real-time PCR (*n*=18, 18, 18, 28, respectively, technical duplicates). Statistical significance was determined by one-way ANOVA followed by the Tukey-Kramer HSD test. **P<0.01 between arbitrary two groups. (**f**,**g**) Heart tissues of Sham- or TAC-operated *Xrcc1*^*f/f*^ and *Xrcc1*^*αMHC-Cre*^ mice were immunostained for CD45 or CD68 (**f**, green). Immunostaining for alpha-actinin (red) was used to label cardiomyocytes. Arrowheads indicate CD45- or CD68-positive cells. Scale bar, 50 μm. The number of CD45- and CD68-positive cells was counted (**g**, *n*=5 each). Statistical significance was determined by one-way ANOVA followed by the Tukey-Kramer HSD test. **P<0.01 between arbitrary two groups. Column and error bars show mean and s.e.m., respectively.

We then investigated whether our *in vitro* findings are also observed *in vivo*. The number of cardiomyocytes positive for phosphorylated H2AX (γH2AX) was increased after TAC operation (Supplementary Fig. 8a,b) and the number was more increased in the heart of *Xrcc1*^*αMHC-Cre*^ mice compared with *Xrcc1*^*f/f*^ mice (Fig. 5a,b). The amount of ROS in the heart was comparable between *Xrcc1*^*αMHC-Cre*^ and *Xrcc1*^*f/f*^ mice (Fig. 5c and Supplementary Fig. 9a,b), suggesting that increased number of γH2AX-positive cardiomyocytes in *Xrcc1*^*αMHC-Cre*^ mice was due to defective SSB repair and SSB accumulation but not overproduction of ROS in *Xrcc1*^*αMHC-Cre*^ mice^27^.

Chromatin immunoprecipitation (ChIP) analyses revealed that binding of NF-κB to the *Vcam1* promoter was increased twofold in TAC-operated *Xrcc1*^*αMHC-Cre*^ cardiomyocytes compared with *Xrcc1*^*f/f*^ cardiomyocytes (Fig. 5d). Expression levels of inflammatory cytokines and infiltration of inflammatory cells were increased in cardiomyocytes after TAC operation, and were more increased in TAC-operated *Xrcc1*^*αMHC-Cre*^ mice compared with *Xrcc1*^*f/f*^ mice (Fig. 5e–g). These *in vivo* results are consistent with our *in vitro* observations and strongly support our hypothesis that accumulation of SSB in cardiomyocytes exacerbates heart failure through the activation of persistent DDR and promoting cardiac inflammation.

### ATM deletion rescues heart failure in Xrcc1-deficient mice

**Figure 6:**
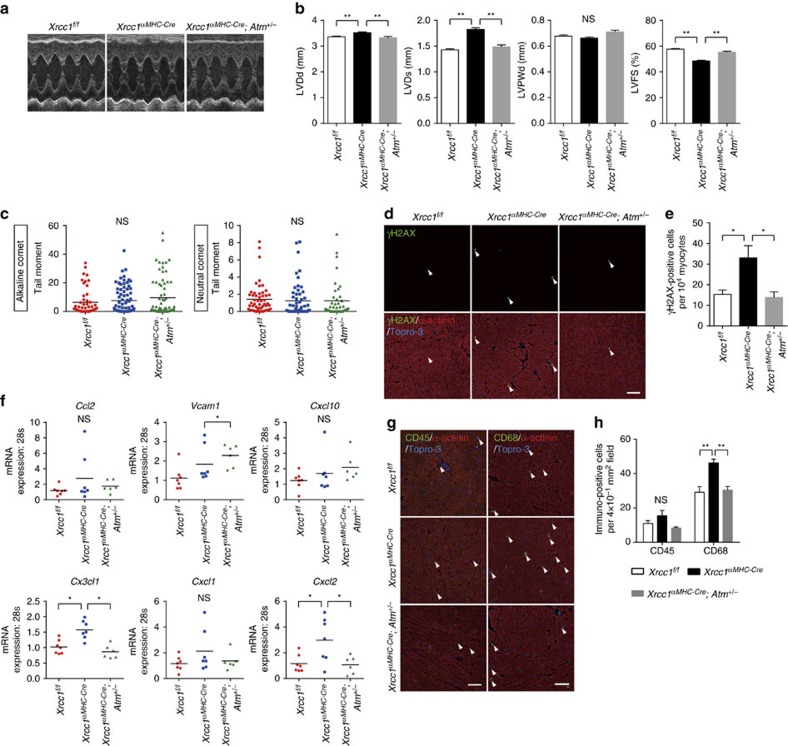
Basal characters of *Xrcc1*^*αMHC-Cre*^ and *Xrcc1*^*αMHC-Cre*^*; Atm*^+/−^ mice. (**a**,**b**) Echocardiographic images (**a**) and cardiac function (**b**) of *Xrcc1*^*f/f*^, *Xrcc1*^*αMHC-Cre*^ and *Xrcc1*^*αMHC-Cre*^*; Atm*^+/−^ mice. Statistical significance was determined by one-way analysis of variance (ANOVA) followed by the Tukey-Kramer HSD test. **P<0.01 between arbitrary two groups. (**c**) The type of DNA damage in cardiomyocytes of *Xrcc1*^*f/f*^, *Xrcc1*^*αMHC-Cre*^ and *Xrcc1*^*αMHC-Cre*^*; Atm*^+/−^ mice was assessed by comet assay (Alkaline comet: *n*=50, 76, 77; Neutral comet: *n*=53, 56, 42, respectively). Statistical significance was determined by Steel-Dwass test. (**d**,**e**) Activation of DDR in *Xrcc1*^*f/f*^, *Xrcc1*^*αMHC-Cre*^ and *Xrcc1*^*αMHC-Cre*^*; Atm*^+/−^ mice was assessed by immunostaining for phosphorylated H2AX (**d**, γH2AX, green, arrowheads). Immunostaining for alpha-actinin (red) was used to label cardiomyocytes. Arrowheads indicate γH2AX-positive cardiomyocytes. Scale bar, 50 μm. The number of γH2AX-positive cardiomyocytes was counted (**e**, *n*=4 each). Statistical significance was determined by one-way ANOVA followed by the Tukey-Kramer HSD test. *P<0.05 between arbitrary two groups. (**f**) The expression levels of inflammatory cytokines in the isolated cardiomyocytes of *Xrcc1*^*f/f*^, *Xrcc1*^*αMHC-Cre*^ and *Xrcc1*^*αMHC-Cre*^*; Atm*^+/−^ mice was assessed by real-time PCR (*n*=7, 7, 6 for each genotype, respectively, technical duplicates). Statistical significance was determined by one-way ANOVA followed by the Tukey–Kramer HSD test. *P<0.05 between arbitrary two groups. (**g**,**h**) Heart tissues of *Xrcc1*^*f/f*^, *Xrcc1*^*αMHC-Cre*^ and *Xrcc1*^*αMHC-Cre*^*; Atm*^+/−^ mice were immunostained for CD45 or CD68 (**g**, green, arrowheads). Immunostaining for alpha-actinin (red) was used to label cardiomyocytes. Arrowheads indicate CD45- or CD68-positive cells. Scale bar, 50 μm. The number of CD45- and CD68-positive cells was counted (**h**, *n*=6, 6, 4 for each genotype, respectively). Statistical significance was determined by one-way ANOVA followed by the Tukey–Kramer HSD test. **P<0.01 between arbitrary two groups. Column and error bars show mean and s.e.m., respectively. LVDd, left ventricular end-diastolic dimension; LVDs, left ventricular end-systolic dimension; LVPWd, left ventricular posterior wall dimension; LVFS, left ventricular fractional shortening (*n*=83, 88, 23 for each genotype, respectively).

To further investigate whether SSB accumulation in cardiomyocytes plays a causative role in the pathogenesis of heart failure through activating DDR, we crossed *Xrcc1*^*αMHC-Cre*^ mice with *Atm*^+/−^ mutants^28^,^29^ to block persistent DDR and obtained *Xrcc1*^*αMHC-Cre*^*; Atm*^+/−^ mice (Supplementary Fig. 10a–d). Mild cardiac dysfunction observed in *Xrcc1*^*αMHC-Cre*^ mice was restored in *Xrcc1*^*αMHC-Cre*^*; Atm*^+/−^ mice (Fig. 6a,b). The level of SSB in cardiomyocytes which tended to be increased in *Xrcc1*^*αMHC-Cre*^ mice remained increased in *Xrcc1*^*αMHC-Cre*^*; Atm*^+/−^ mice (Fig. 6c and Supplementary Fig. 11a,b), however, the number of γH2AX-positve cardiomyocytes that was increased in *Xrcc1*^*αMHC-Cre*^ mice was reduced to the level of *Xrcc1*^*f/f*^ mice in the heart of *Xrcc1*^*αMHC-Cre*^*; Atm*^+/−^ mice (Fig. 6d,e), suggesting that heterozygous *Atm* gene deletion indeed suppressed DDR activation which was induced by DNA SSB. Expression levels of inflammatory cytokines in cardiomyocytes (Fig. 6f) and infiltration of inflammatory cells (Fig. 6g,h) were mildly increased in the heart of *Xrcc1*^*αMHC-Cre*^ mice and were attenuated in *Xrcc1*^*αMHC-Cre*^*; Atm*^+/−^ mice with the exception of *Cxcl10* and *Vcam1* gene expression. There was an apparent discrepancy between the level of SSB, which tended to increase but did not reach statistical significance, and the other cardiac phenotypes in *Xrcc1*^*αMHC-Cre*^ mice. This discrepancy may be explained by the presence of threshold between the level of DNA SSB and the downstream events in cardiomyocytes. Just like there is a threshold between the level of DNA damage and cell death (cell death is triggered when the level of DNA damage is above the threshold), it is conceivable to think that persistent DDR and the following inflammatory program are triggered when the level of unrepaired SSB in each cell is above the threshold.

**Figure 7:**
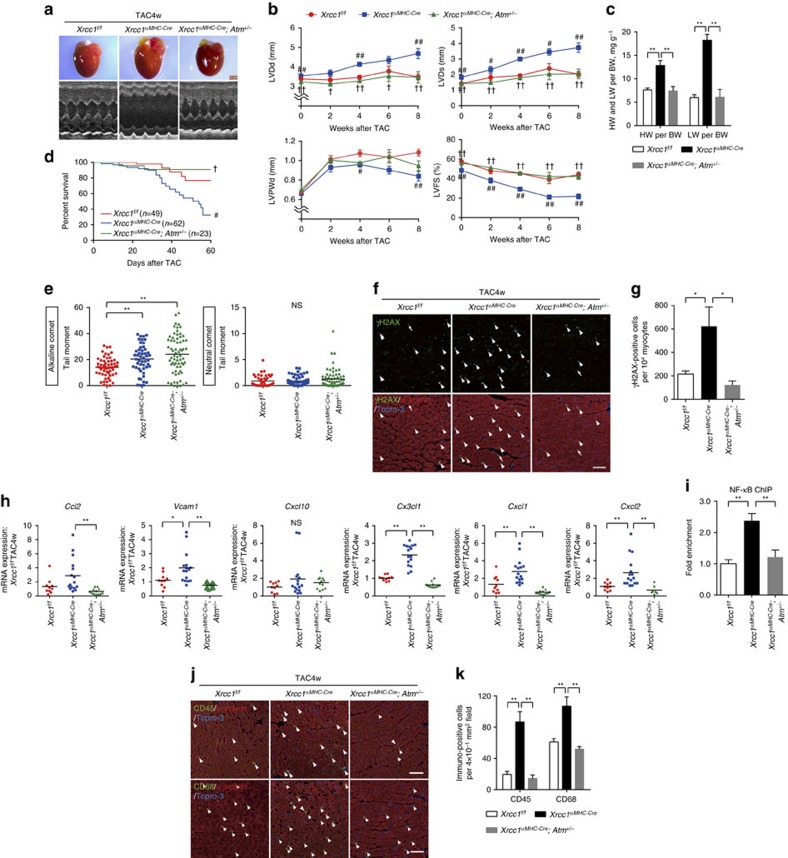
*ATM* gene deletion rescues the cardiac phenotypes of *Xrcc1* deficient mice. (**a**,**b**) Macroscopic and echocardiographic images (**a**) and cardiac function (**b**) of TAC-operated *Xrcc1*^*f/f*^, *Xrcc1*^*αMHC-Cre*^ and *Xrcc1*^*αMHC-Cre*^*; Atm*^+/−^ mice (*Xrcc1*^*f/f*^mice: *n*=83, 21, 46, 11, 27; *Xrcc1*^*αMHC-Cre*^ mice: *n*=88, 28, 60, 13, 16; *Xrcc1*^*αMHC-Cre*^*; Atm*^+/−^ mice: *n*=28, 22, 22, 7, 7 at each time point, respectively). Scale bar, 2 mm. (**c**) Heart, lung, and body weight of TAC-operated *Xrcc1*^*f/f*^, *Xrcc1*^*αMHC-Cre*^ and *Xrcc1*^*αMHC-Cre*^*; Atm*^+/−^ mice were weighed 8 weeks after the surgery (*n*=8, 5, 6 for each genotype, respectively). (**d**) Survival curves of TAC-operated *Xrcc1*^*f/f*^, *Xrcc1*^*αMHC-Cre*^ and *Xrcc1*^*αMHC-Cre*^*; Atm*^+/−^ mice (*n*=49, 62, 23, respectively). (**e**–**k**) TAC-operated *Xrcc1*^*f/f*^, *Xrcc1*^*αMHC-Cre*^ and *Xrcc1*^*αMHC-Cre*^*; Atm*^+/−^ mice were analysed 4 weeks after the surgery. The type of DNA damage in cardiomyocytes was assessed by comet assay (**e**, Alkaline comet: *n*=50, 76, 77; Neutral comet: *n*=53, 56, 42, respectively). Activation of DDR was assessed by immunostaining for phosphorylated H2AX (**f**, γH2AX, green, arrowheads). Arrowheads indicate γH2AX-positive cardiomyocytes and arrows indicate γH2AX-positive non-cardiomyocytes. Scale bar, 50 μm. The number of γH2AX-positive cardiomyocytes was counted (**g**, *n*=4 each). Expression levels of inflammatory cytokines in the isolated cardiomyocytes were assessed by real-time PCR (**h**, *n*=10, 16, 12 for each genotype, respectively, technical duplicates). ChIP–qPCR analysis of binding of NF-κB to the *Vcam1* promoter region. Data is presented as fold enrichment relative to TAC-operated *Xrcc1*^*f/f*^ mice (**i**, *n*=4, 5, 5, respectively). Heart tissues were immunostained for CD45 or CD68 (**j**, green, arrowheads). Arrowheads indicate CD45- or CD68-positive cells. Scale bar, 50 μm. The number of CD45- and CD68-positive cells was counted (**k**, *n*=4 each). Statistical significance was determined by one-way analysis of variance followed by the Tukey–Kramer HSD test for (**b**) (at each time point), (**c**,**g**,**h**,**i**,**k**), by Wilcoxon test for **d** and by Steel–Dwass test for **e**, ^#^*P*<0.05; ^##^*P*<0.01 between *Xrcc1*^*f/f*^ and *Xrcc1*^*αMHC-Cre*^ mice. †*P*<0.05; ^††^*P*<0.01 between *Xrcc1*^*αMHC-Cre*^ and *Xrcc1*^*αMHC-Cre*^*; Atm*^+/−^ mice. *P<0.05; **P<0.01 between arbitrary two groups. Column and error bars show mean and s.e.m., respectively.

We finally performed the TAC surgery to these mice and found that heterozygous *Atm* gene deletion attenuated the progression of severe left ventricular dysfunction (Fig. 7a,b), alleviated the signs of heart failure (Fig. 7c) and improved the mortality in *Xrcc1*^*αMHC-Cre*^ mice (Fig. 7d). The level of SSB in cardiomyocytes remained high in *Xrcc1*^*αMHC-Cre*^*; Atm*^+/−^ mice, however, the number of γH2AX-positve cardiomyocytes was reduced to the level of *Xrcc1*^*f/f*^ mice (Fig. 7e-g), suggesting that heterozygous *Atm* gene deletion suppressed DDR activation also after the TAC operation. The expression level of inflammatory cytokines in cardiomyocytes (Fig. 7h), binding of NF-κB to the *Vcam1* promoter (Fig. 7i) and infiltration of inflammatory cells (Fig. 7j,k) were also attenuated in *Xrcc1*^*αMHC-Cre*^*; Atm*^+/−^ mice compared with *Xrcc1*^*αMHC-Cre*^ mice. These results collectively suggest that SSB-induced activation of DDR plays a causal role in cardiac inflammation and heart failure in response to pressure overload.

## Discussion

**Figure 8:**
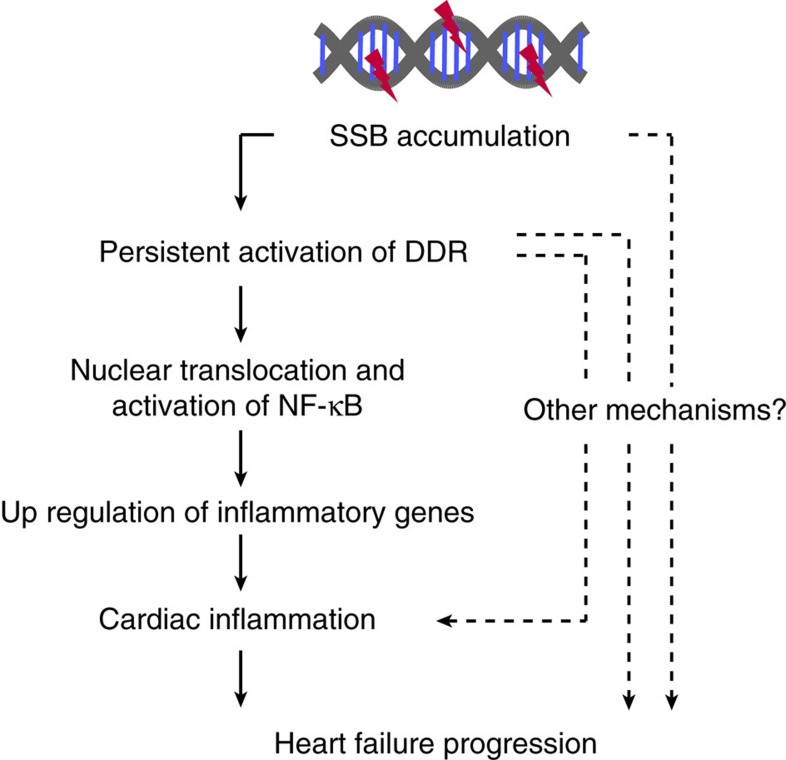
Possible roles of SSB accumulation in pathogenesis of heart failure. Accumulation of DNA SSB in cardiomyocytes induces persistent activation of DDR and subsequent activation of NF-κB pathway, resulting in increased expressions of inflammatory cytokines. These mechanisms may contribute, at least in part, to increased cardiac inflammation and the progression of pressure overload-induced heart failure.

Accumulation of unrepaired oxidative DNA damage and activation of DDR have been observed in the failing heart^5^,^6^,^30^, however, their roles in the pathogenesis of heart failure remain elusive. In the present study, we identified the type of DNA damage which triggers DDR in cardiomyocytes and demonstrated that activation of DDR contributes to, at least in part, cardiac inflammation in the failing heart. We found that unrepaired DNA SSB accumulates in cardiomyocytes of pressure overload-induced heart failure model mice. Accumulation of DNA SSB induced persistent DDR and upregulated the expression of inflammatory cytokines through NF-κB pathway. Accumulation of DNA SSB was more increased in the mice lacking XRCC1, an essential protein for SSB repair. Increased level of SSB accumulation was associated with more activation of DDR, expression of inflammatory cytokines and infiltration of inflammatory cells, and also with exacerbation of cardiac dysfunction after pressure overload. Worse cardiac phenotype that was observed in the mice with defective SSB repair was rescued by blocking DDR activation through *Atm* gene deletion collectively suggesting the important role of SSB accumulation and subsequent activation of DDR in the pathogenesis of heart failure (Fig. 8).

In the present manuscript, we showed that accumulation of unrepaired SSB plays a causative role in the pathogenesis of heart failure. Unrepaired SSB develops into DNA DSB during mitosis^14^ in mitotic cells, therefore, accumulation of SSB is supposed to be specific to post-mitotic cells such as neurons or cardiomyocytes^15^. Accumulation of unrepaired SSB in neural tissue is observed in spinocerebellar ataxia with axonal neuropathy 1 (SCAN1)^15^. Mutation of SSB repair enzyme, Tyrosyl-DNA phosphodiesterase 1, is observed in the patients of SCAN1 (refs 31, 32) and DNA SSB is accumulated in the neural tissue of *Tdp1*^*−/−*^ mice^33^. Our findings indicate that SSB accumulation also occurs in stressed, post-mitotic cells and plays pathogenic role in non-genetic disorders. Compared with the pathogenic role of DSB, knowledge about the roles of SSB in human diseases is limited. Future study may reveal the role of SSB in the pathogenesis of various genetic or non-genetic diseases.

ATM becomes activated in response to DNA damage to stop the cell cycle and promote DNA damage repair. Its activation is essentially protective for the organism to maintain homeostasis. In the present study, however, we show that ATM may also play a detrimental role in pressure overload-induced heart failure through NF-κB-dependent induction of inflammatory gene expression. Recent reports also suggested that aberrant activation of ATM plays detrimental roles in myocardial infarction model mice, that is, ATM promotes cardiac inflammation during myocardial ischemia^34^ and cardiac remodelling after myocardial infarction are attenuated in ATM knockout mice^34^,^35^. Further investigations for the precise molecular action of ATM against chronic DNA damage may provide a novel therapeutic target for heart failure.

On DNA SSB, PARP-1 is rapidly recruited to the damaged site, where it synthesizes branched ADP-ribose polymers (pADPr) on SSB repair proteins including PARP-1, XRCC1 and DNA ligases and facilitates SSB repair^13^,^36^. It was surprising for us to see that protein poly(ADP-ribosylation) and the expression levels of the genes that work for SSB repair were decreased in the failing heart (Fig. 1g–i), despite the production of ROS and the amount of DNA damage was increased (Fig. 1a,b,j–l), suggesting that decreased SSB repair activity as well as increased ROS production may play a role in SSB accumulation in the heart after TAC operation (Supplementary Fig. 3). We do not know, at the moment, why SSB repair system become downregulated in the failing heart, but downregulation of SSB repair system may have a role in preventing cardiomyocyte death against massive DNA damage. Since the source of ADP-ribose to form pADPr is nicotinamide adenine dinucleotide (NAD^+^), hyperactivation of PARP-1 on excess DNA damage consumes the intracellular NAD^+^ pool, leading to exhaustion of ATP and necrotic death^13^. Hyperactivation of PARP-1 is also reported to induce caspase-independent cell death termed parthanatos^37^,^38^. Although the system for eliminating the cells with massive DNA damage is beneficial to prevent cancer formation, it might become critical for the tissue consisted of post-mitotic cells such as cardiomyocytes. Cardiomyocytes may have intrinsic mechanism to downregulate DNA damage repair system for cell (and organ) survival, at the expense of DNA damage accumulation.

In summary, we identified a previously unrecognized role of DNA SSB in the pathogenesis of heart failure. Accumulation of SSB in cardiomyocytes activated DDR and persistent DDR promoted cardiac inflammation in pressure overload-induced heart failure. From a clinical perspective, approaches that promote efficient SSB repair or suppress aberrant DDR may become a novel therapeutic strategy against heart failure.

## Methods

### Animal models

All animal procedures were approved by the Institutional Animal Care and Use Committee of Osaka University (22-056). C57BL/6 mice were purchased from CLEA JAPAN and α*-MHC-Cre*^*tg*^ mice were from Jackson Laboratory (stock#009074, stock name: STOCK Tg (Myh6-cre)1Jmk/J)^19^. *Xrcc1*^*flox/flox*^ mice^18^ were backcrossed to C57BL/6 for six generations before the experiments. Conditional deletion of *Xrcc1* in cardiomyocytes was achieved by crossing *Xrcc1*^*flox/flox*^ homozygous mice with α*MHC-Cre*^*tg*^ hemizygous mice. *Xrcc1*^*flox/flox*^; α*MHC-Cre*^*tg*^ (*Xrcc1*^*αMHC-Cre*^) mice were used as cardiomyocyte-specific *Xrcc1* knockout mice. Genotyping PCR was performed as previously described^18^. In brief, deletion of *Xrcc1* allele in the heart was examined by detection of floxed allele (274 bp) using primers 5′-TATGCTTGCTGTACAGGGATTGGGC-3′ and 5′-TGGACCATGAAAAAGCTGTGTGC-3′.

To generate cardiomyocyte-specific *Xrcc1* knockout mice with an *Atm*^*+/−*^ background, *Xrcc1*^*αMHC-Cre*^ mice were crossed with *Atm*^*+/−*^ mice (Jackson Laboratory; stock#0008536, stock name: B6.129S6-*Atm*^*tm1Awb*^/J) and cardiomyocyte-specific *Xrcc1* knockout mice with systemic loss of one allele of ATM (*Xrcc1*^*αMHC-Cre*^*; Atm*^+/−^ mice) were obtained. Genotyping PCR was performed according to the protocol from Jackson Laboratory. Expected PCR product sizes of *Atm* wild-type and mutant allele were 147 and 280 bp, respectively.

### TAC operation and echocardiography

TAC surgery was performed to 10- to 12-week-old male mice^39^. The transverse aorta was constricted with a 7-0 silk suture paralleled with a 27-gauge blunt needle, which was removed after constriction. Sham-operated animals, which underwent a similar surgical procedure without aortic constriction, were used as controls. TAC surgery was performed to the mice randomly selected from those weighing 25–30 g at the age of 10–12 weeks. Surgeon was not informed about the genotypes of the mice. The mice which died within 1 day after the operation were excluded from the analysis. Transthoracic echocardiography was performed on conscious mice with a Vevo 770 imaging system (Visualsonics, Inc.). M-mode echocardiographic images were obtained from a longitudinal view to measure the LV size and LV function.

### Cell culture and siRNA transfections

Hearts were excised from newborn (1- to 2-day-old) Sprague-Dawley rats and neonatal rat cardiomyocytes (NRCMs) were isolated using Neonatal Cardiomyocyte Isolation System (Worthington Biochemical Corp.) according to the manufacturer's instructions^40^. NRCMs were cultured in DMEM (Wako) supplemented with 10% fetal bovine serum, 10 μM cytosine β-D-arabinofuranoside hydrochloride (Ara-C, Sigma). Ara-C was supplemented to inhibit the proliferation of non-cardiomyocytes and we confirmed that it does not affect DNA strand breaks in NRCMs (Supplementary Fig. 12). DNA damage was induced by irradiation (20 Gy), methyl methanesulfonate treatment (Sigma, 0.05 mg ml^−1^ for 10 min) and Doxorubicin (Sigma, 1 μM for 3 h). For gene silencing, NRCMs were transfected with pre-designed stealth RNAi siRNA (Invitrogen) using Lipofectamine RNAiMAX Transfection Reagent (Invitrogen) according to the manufacturer's instructions. The scrambled oligonucleotide was used as a negative control. Sequences were as follows:

*Xrcc1* siRNA sequence:

5′-AGCCAUACAGCAAGGACUCACCCUA-3′ (sense);

5′-UAGGGUGAGUCCUUGCUGUAUGGCU-3′ (antisense).

*Atm* siRNA sequence:

5′-UUCUCUUGCAAUCUCAUCAGGACGC-3′ (sense);

5′-GCGUCCUGAUGAGAUUGCAAGAGAA-3′ (antisense).

### Measurement of ROS production

To measure ROS production, heart samples were immediately embedded in Tissue-Tek OCT cryo-embedding compound (Miles Laboratories) and stored at −80 °C. Unfixed frozen samples were cut into 5 μm-thick sections and placed on glass slides. Tissue section was covered with dihydroethidium (DHE, Sigma, 10 μM) and incubated in a light-protected humidified chamber for 30 min at 37 °C. Ethidium fluorescence (excitation at 490 nm, emission at 610 nm) was examined by fluorescent microscopy and mean fluorescence intensity was measured by using ImageJ software.

To measure the level of hydrogen peroxide, mice hearts were excised and cut into small pieces. After the incubation in Krebs-HEPES buffer (128 mM NaCl, 2.5 mM KCl, 1 mM MgSO_4_, 2.7 mM CaCl_2_, 16 mM D-glucose, 20 mM HEPES), hydrogen peroxide levels per tissue weight were measured using Amplex red assay (Invitrogen) according to the manufacturer's instructions.

### Comet assay

DNA strand breaks were evaluated by single cell electrophoresis (comet assay)^10^. The heart was quickly excised after deep anaesthesia and mounted onto a Langendorff perfusion apparatus^41^. The hearts were perfused with calcium-free buffer (0-Ca^2+^ buffer: 126 mM NaCl, 8.8 mM KCl, 2.0 mM MgCl_2_, 24 mM HEPES, 5.0 mM sodium pyruvate, 11 mM D-glucose, 1 × Glutamax, 30 mM 2,3-Butanedione monoxime, 4.4 mM creatine monohydrate, 8.0 mM Taurine, pH 7.4) for 5 min at 2 ml min^−1^, then with collagenase solution (1 mg ml^−1^ type 2 collagenase, 0.2 mg ml^−1^ protease type 14, 50 μM CaCl_2_ in 0-Ca^2+^ buffer) for 10 min at 3 ml min^−1^. The whole system was maintained at 39 °C. Following perfusion, hearts were placed in a 60-mm dish containing 50 μM Ca^2+^ buffer (50 μM CaCl_2_ in 0-Ca^2+^ buffer) and minced with micro-scissors into small pieces. Non-cardiomyocytes were quickly removed according to their size (smaller than cardiomyocytes) using cell strainer^42^. Purity of cardiomyocytes was ∼90% (Supplementary Fig. 13a,b). Thirty-microliter of the cell suspension containing 600 cardiomyocytes were mixed with 90 μl of 1% low melting point agar, transferred onto FLARE Slides (Trevigen), and incubated for 30 min at 4 °C. For alkaline comet assay, cells were lysed in alkaline buffer (1.2 M NaCl, 100 mM EDTA, 0.1% sodium lauryl sarcosinate, pH 13.1) for 45 min at 4 °C and electrophoresed for 25 min in 0.03 M NaCl and 2 mM EDTA at 12 V and 24 mA constant current. For neutral comet assay, cells were lysed in neutral buffer (1.2 M NaCl, 100 mM EDTA, 10 mM Tris, 1% sodium lauryl sarcosinate, 0.5% TritonX-100, 10% dimethylsulfoxide, pH 9.5) for 1.5 h at 4 °C and electrophoresed for 25 min in TBE buffer at 12 V and 4 mA constant current. Cells were then stained with SYBR Green (Life Technologies). Comet tail moment (=tail length × tail % DNA/100) of each cell was calculated using Comet Score analysis software (TriTek Corp.). The same procedure was performed to measure DNA strand breaks in cultured NRCMs except the lysis duration. NRCMs were lysed for 2 h at 4 °C in alkaline buffer and also lysed for 2 h at 4 °C in neutral buffer. Experimental condition of the comet assay was determined using positive and negative controls (Supplementary Figs 2a, 6a).

### Immunofluorescence staining

For immunofluorescent staining, hearts were excised and immediately embedded in Tissue-Tek OCT cryo-embedding compound (Miles Laboratories). Cryostat sections at 4 μm were fixed in acetone and incubated with primary antibodies over night after blocking with 5% normal donkey serum. For mice primary antibodies, blocking was performed using Mouse on Mouse (M.O.M) basic kit (Vector Laboratories). After washing with PBS, samples were stained with appropriate secondary antibodies (anti-rabbit IgG-Alexa 488 1:200, anti-rat IgG-Alexa 488 1:200, streptavidin-Alexa 488 1:200 and streptavidin-Alexa 594 1:200, Molecular Probes) for 1 h. Membrane and the nuclei of the cells were counterstained with Wheat Germ Agglutinin-Alexa 594 (Molecular Probes, 1:200) and TO-PRO-3 iodide 642/661 (Molecular Probes, 1:200), respectively.

*In situ* oligo ligation staining was performed with ApopTag Peroxidase *In Situ* Oligo Ligation (ISOL) Kit (Millipore) according to the manufacturer's instructions. The biotinylated oligonucleotide that labels DNA strand breaks with blunt ends was used. We used streptavidin-Alexa 488 instead of streptavidin-peroxidase for fluorescent labelling of ISOL positive cells. For immunofluorescent staining of the cultured cells, cells cultured on gelatin-coated coverslips were fixed with 4% paraformaldehyde and then permeabilized with 0.25% Triton-X100. Images were obtained by the confocal laser scanning microscopy LSM700 (Carl Zeiss).

Primary antibodies for immunofluorescent staining were as follows: rabbit polyclonal anti-NBS1 antibody (ab23996, Abcam, 1:100), mouse monoclonal anti-Poly ADP-ribose antibody (clone 10H, BD Biosciences, 1:500), mouse monoclonal anti- NF-κB antibody (clone L8F6, Cell Signaling Technology, 1:400), mouse monoclonal anti-CD45 antibody (clone 30-F11, RD, 1:50), rat monoclonal anti-mouse CD68 antibody (clone FA-11, AbD Serotec, 1:100), rabbit polyclonal anti-γH2AX (phospho-Ser139) antibody (ab2893, Abcam, 1:500), and mouse monoclonal anti-Sarcomeric Alpha Actinin (clone EA-53, Abcam, 1:100).

### Western blotting

Heart tissue or cultured cardiomyocytes were homogenized and lysed with RIPA buffer with protease and phosphatase inhibitor cocktails for 30 min on ice. Lysates were centrifuged at 15,000*g* for 20 min and the supernatants were used as whole-cell extracts. For nuclear fractionation, samples were homogenized and lysed in hypotonic buffer (10 mM HEPES, 1.5 mM MgCl_2_, 10 mM KCl, 0.5 mM DTT and 0.05% NP-40, pH 7.9) with protease and phosphatase inhibitor cocktails for 5 min on ice. The extracts were centrifuged at 800*g* for 10 min and the pellet were lysed in RIPA buffer to obtain nuclear extracts. The protein samples were subjected to SDS–PAGE and transferred to nitrocellulose membranes (GE Healthcare). Membranes were incubated with primary antibodies, followed by horseradish peroxidase-conjugated anti-mouse, anti-rabbit, anti-goat, rabbit trueblot anti-rabbit, mouse trueblot anti-mouse IgG (Jackson ImmunoResearch and ebioscience). Immunoreactive signals were detected with the ECL Plus Western Blotting Detection System (GE Healthcare).

Following primary antibodies were used for western blotting: rabbit polyclonal anti-XRCC1 antibody (#2735, Cell Signaling Technology, 1:1,000), mouse monoclonal anti-phosphorylated ATM (Ser1981) antibody (10H11.E12, Rockland, 1:1,000), rabbit polyclonal anti-ATM antibody (H-248, Santa Cruz, 1:500 for *in vitro* experiments and clone MAT3-4G10/8, Abcam, 1:10,000 for *in vivo* experiments), rabbit polyclonal anti-γH2AX (Phospho-Ser139) antibody (ab2893, Abcam, 1:1,000), rabbit polyclonal anti-Histone H2AX antibody (ab11175, Abcam, 1:5,000), rabbit polyclonal anti-phosphorylated p53 (Ser15) antibody (#9284, Cell Signaling Technology, 1:1,000), mouse monoclonal anti-p53 antibody (clone 1C12, Cell Signaling Technology, 1:1,000) and HRP-conjugated anti-GAPDH antibody (ab9482, Abcam, 1:15,000). Uncropped immunoblot images are presented in Supplementary Fig. 14.

### RNA analysis

Total RNA was extracted using TRIzol reagents (Invitrogen) for heart tissues or PureLink RNA Mini Kit (Ambion) for cultured cells according to manufacturer's instructions. RNA samples were subjected to DNase treatment to remove genomic DNA using TURBO DNA-free Kit (Ambion) or PureLink DNase Set (Ambion) and were reverse-transcribed using SuperScript VILO cDNA Synthesis Kit (Invitrogen). Quantitative real-time PCR was performed using Universal Probe Library (UPL, Roche) and Light Cycler TaqMan Master kit (Roche). Relative expression levels of the target genes were normalized to the expression of internal control gene using comparative Ct method. Primer sequences and the corresponding UPL numbers were designed with online program provided by Roche.

### ChIP–qPCR

Isolated cardiomyocytes were crosslinked with 0.3% formaldehyde at room temperature for 10 min. Samples were sonicated using Bioruptor Plus (Diagenode). After sonication, soluble chromatin was incubated with an anti- NF-κB antibody (5 μg, sc-372X, Santa Cruz). Specific immunocomplexes were precipitated with Dynabeads Protein G (Life Technologies). Immunoprecipitates were washed, reverse-crosslinked and purified by Wizard SV Gel and PCR Clean-Up System (Promega). Extracted DNA was used for quantitative PCR (qPCR). Fold enrichment was determined as the fold change in per cent input (ChIP signal/input signal) at the target region compared to the control region. Primers were designed to amplify the *Vcam1* promoter region containing putative NF-κB-binding sites. The control primers were designed against non-conserved and non-repetitive sequences at 25 kbp upstream of *Vcam1* gene. Quantitative real-time PCR was performed using Universal Probe Library (UPL, Roche) and Light Cycler TaqMan Master kit (Roche). Primer sequences were as follows: target region: 5′-TAGAGAGGCGGAGGGAAATC-3′ and 5′-GCTTCATGATGGCAAGTGG-3′; the control region: 5′-GAAATCATCATTTGTGTATCTCGAA-3′ and 5′-AGGGCTTGTTGATTCTGCTC-3′.

### Statistical analysis

All values are presented as mean±s.e.m. Two-group comparison was analysed by unpaired two-tailed Student's *t*-test or Mann–Whitney *U*-test. Multiple group comparison was performed with one-way analysis of variance followed by the Tukey–Kramer HSD test or Steel-Dwass test for comparison of arbitrary two groups. Survival curves after the TAC surgery were analysed by Kaplan–Meier method and comparison of the two groups was performed with Wilcoxon test. Significant differences were defined as *P*<0.05. The optimal sample size (*n*=26) to detect a difference of survival rate after pressure overload between *Xrcc1*^*f/f*^ and *Xrcc1*^*αMHC-Cre*^ mice was calculated based on our preliminary data that indicate the median survival time of TAC-operated *Xrcc1*^*αMHC-Cre*^ mice is 60 days. Since the actual median survival time of TAC-operated *Xrcc1*^*αMHC-Cre*^ mice in the present study was 54 days, we reduced the optimal sample size of *Xrcc1*^*αMHC-Cre*^*; Atm*^+/−^ mice (*n*=23). We used a power of 80% and a type I error probability of 0.05. Sample size calculations were performed using the software Power and Sample Size Calculations. All the statistical analyses were reviewed by Professor Ayumi Shintani, a statistical expert at Department of Clinical Epidemiology and Biostatistics, Osaka University, Japan.

### Data availability

The data that support the findings of this study are available from the corresponding author on reasonable request.

## Additional information

**How to cite this article:** Higo, T. *et al*. DNA single-strand break-induced DNA damage response causes heart failure. *Nat. Commun.* **8,** 15104 doi: 10.1038/ncomms15104 (2017).

**Publisher's note:** Springer Nature remains neutral with regard to jurisdictional claims in published maps and institutional affiliations.

## Supplementary Material

Supplementary Information Supplementary Figures and Supplementary Reference.
