# Supplementary material for: DNA single-strand break-induced DNA damage response causes heart failure
Source: Nat Commun. 2017 Apr 24;8:15104. doi: 10.1038/ncomms15104 (PMC5413978; doi:10.1038/ncomms15104)
Supplement: Supplementary Information — Supplementary Figures and Supplementary Reference. [file ncomms15104-s1.pdf]

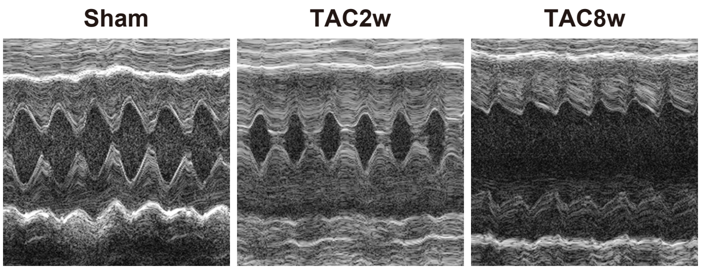

Supplementary Figure 1. Representative echocardiogram of Sham- and TAC-operated mice. Left ventricular hypertrophy was observed in 2 weeks (TAC2w), and left ventricular dysfunction was observed in 8 weeks (TAC8w) after the TAC surgery.

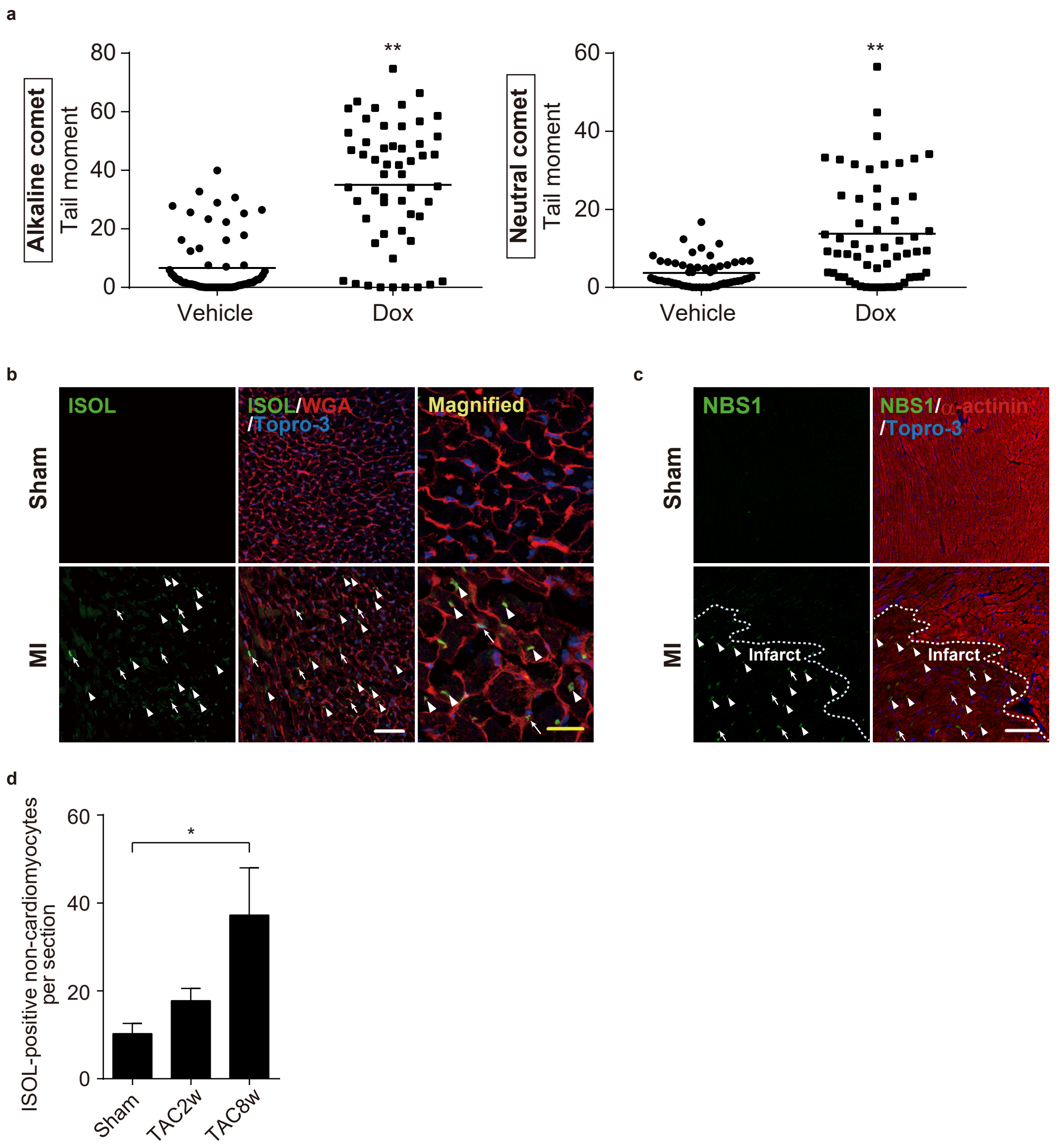

Supplementary Figure 2. Detection of DNA DSB in cardiomyocytes and non-cardiomyocytes of the mice heart. a, Mice were treated with doxorubicin (intraperitoneal injection, 20 mg kg^{-1}) to induce DNA DSB. Cardiomyocytes were isolated 24 h after the treatment and analyzed by comet assay (Alkaline comet: n=67, 54; Neutral comet: 54, 58, respectively). Statistical significance was determined by Mann-Whitney *U* test. **p<0.01 versus Vehicle. **b, c,** Coronary artery ligation was performed to produce myocardial infarction and induce cardiomyocyte apoptosis. Mice were sacrificed 16 h after coronary artery ligation and ISOL staining (**b,** green) and immunostaining for NBS1 (**c,** green) were performed as described in Methods section. ISOL- or NBS1-positive cells were stained green. Wheat germ agglutinin (WGA, red) or immunostaining for alpha-actinin (red) were used to visualize cardiomyocytes. Arrowheads indicate ISOL- or NBS1-positive cardiomyocytes and arrows indicate ISOL-positive non-cardiomyocytes. White scale bar, 50 µm; yellow scale bar, 20 µm. **d,** Fragmented DNA and DSB were labeled with ISOL staining as described in Figure. 1c and the number of ISOL-positive non-cardiomyocytes was counted (n=4 each). Statistical significance was determined by One-way ANOVA followed by the Tukey-Kramer HSD test. *p<0.05 between arbitrary two groups. Column and error bars show mean and s.e.m, respectively.

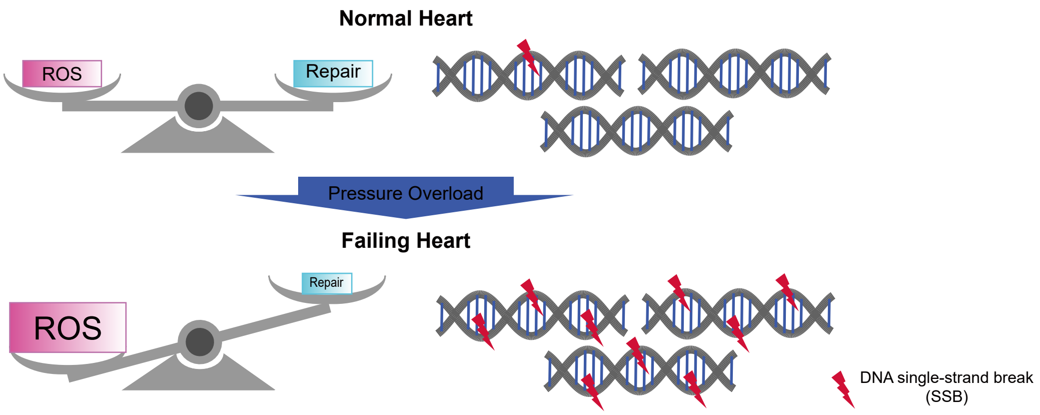

Supplementary Figure 3. Possible Mechanism of SSB accumulation in the failing heart. Down-regulation of SSB repair as well as increased ROS may account for SSB accumulation in the failing heart. ROS: reactive oxygen species.

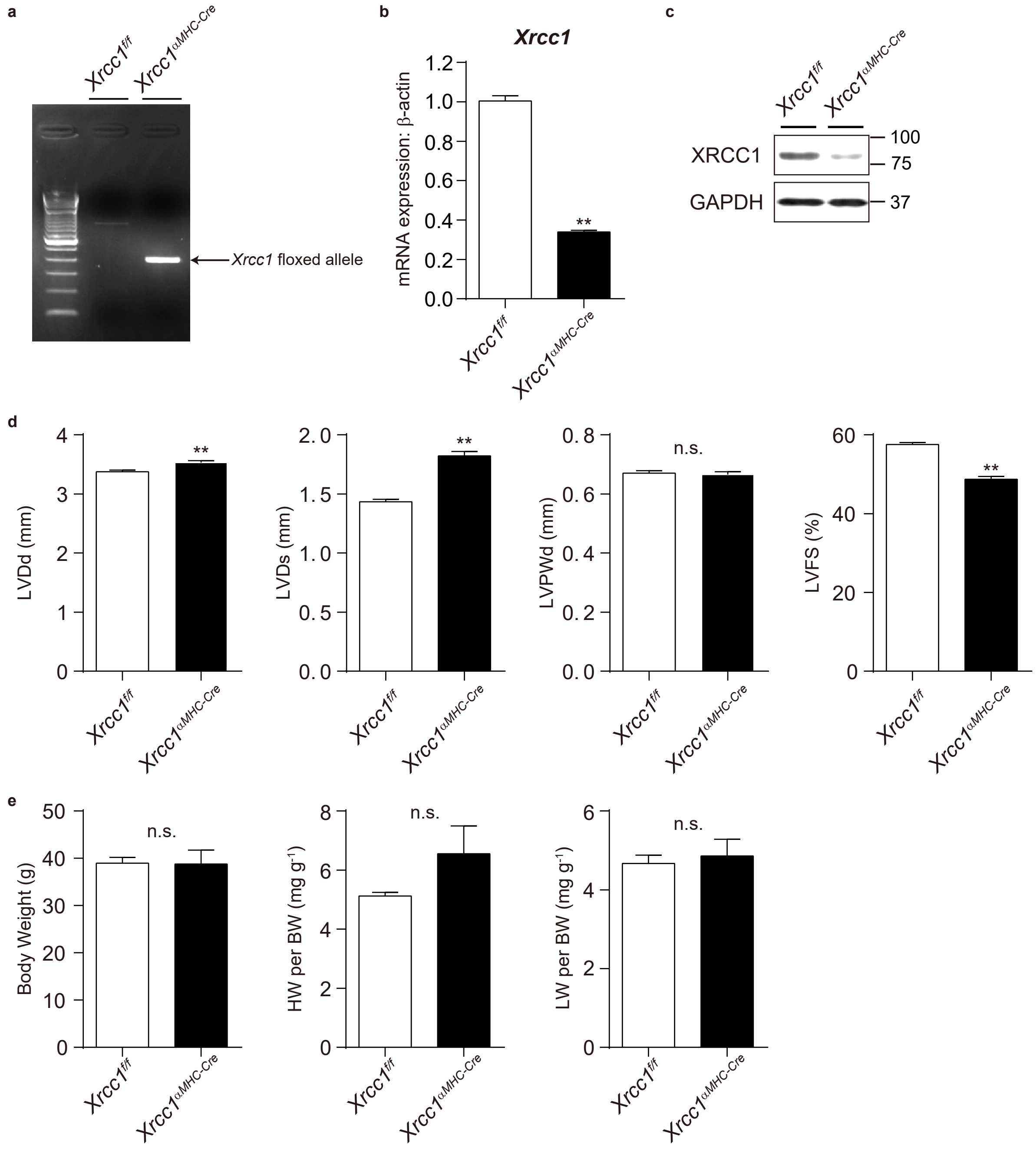

Supplementary Figure 4. Basal characterization of *Xrcc1*$^{αMHC-Cre}$ mice. a-c, Efficient deletion of Xrcc1 in the heart of *Xrcc1*$^{αMHC-Cre}$ mice was confirmed at DNA, RNA, and protein level by genotyping PCR (**a**), real-time PCR (**b,** n=13, 18, technical duplicates) and western blotting (**c**), respectively. Statistical significance was determined by Student's *t* test for **b**. **p<0.01 versus *Xrcc1*$^{f/f}$ mice.**d,** Cardiac function of *Xrcc1*$^{f/f}$ and *Xrcc1*$^{αMHC-Cre}$ mice was assessed by echocardiogram. LVDd, LV end-diastolic dimension; LVDs, LV end-systolic dimension; LVPWd, LV posterior wall dimension, LVFS, LV fractional shortening (n=80, 85 for each genotype). Statistical significance was determined by Student's *t* test. **p<0.01 versus *Xrcc1*$^{f/f}$ mice.**e**, Heart, lung, and body weight of 1-year-old *Xrcc1*$^{f/f}$ and *Xrcc1*$^{αMHC-Cre}$ mice were weighed (n=8 each). Statistical significance was determined by Student's *t* test for body weight and LW/BW and by Mann-Whitney *U* test for HW/BW. Column and error bars show mean and s.e.m, respectively.

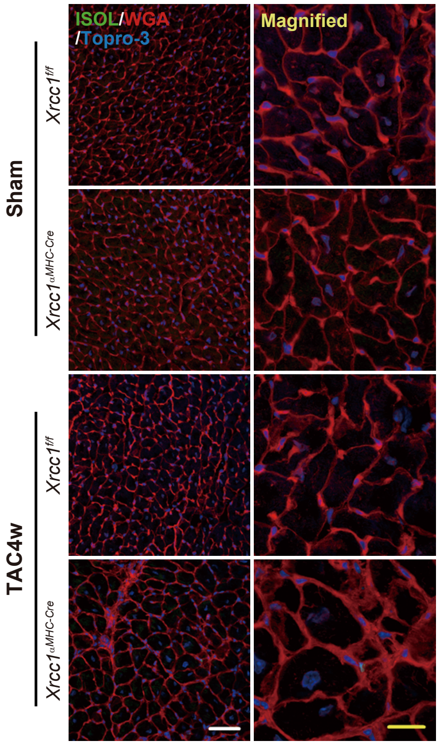

Supplementary Figure 5. Loss of Xrcc1 in cardiomyocytes does not increase DNA DSB in mice. Fragmented DNA and DSB in cardiomyocytes of Sham- or TAC-operated *Xrcc1*$^{f/f}$ and *Xrcc1*$^{\alpha MHC\text{-}Cre}$ mice were labeled by ISOL staining (green). Wheat germ agglutinin (WGA, red) was used to visualize cardiomyocytes. White scale bar, 50 µm; yellow scale bar, 20 µm.

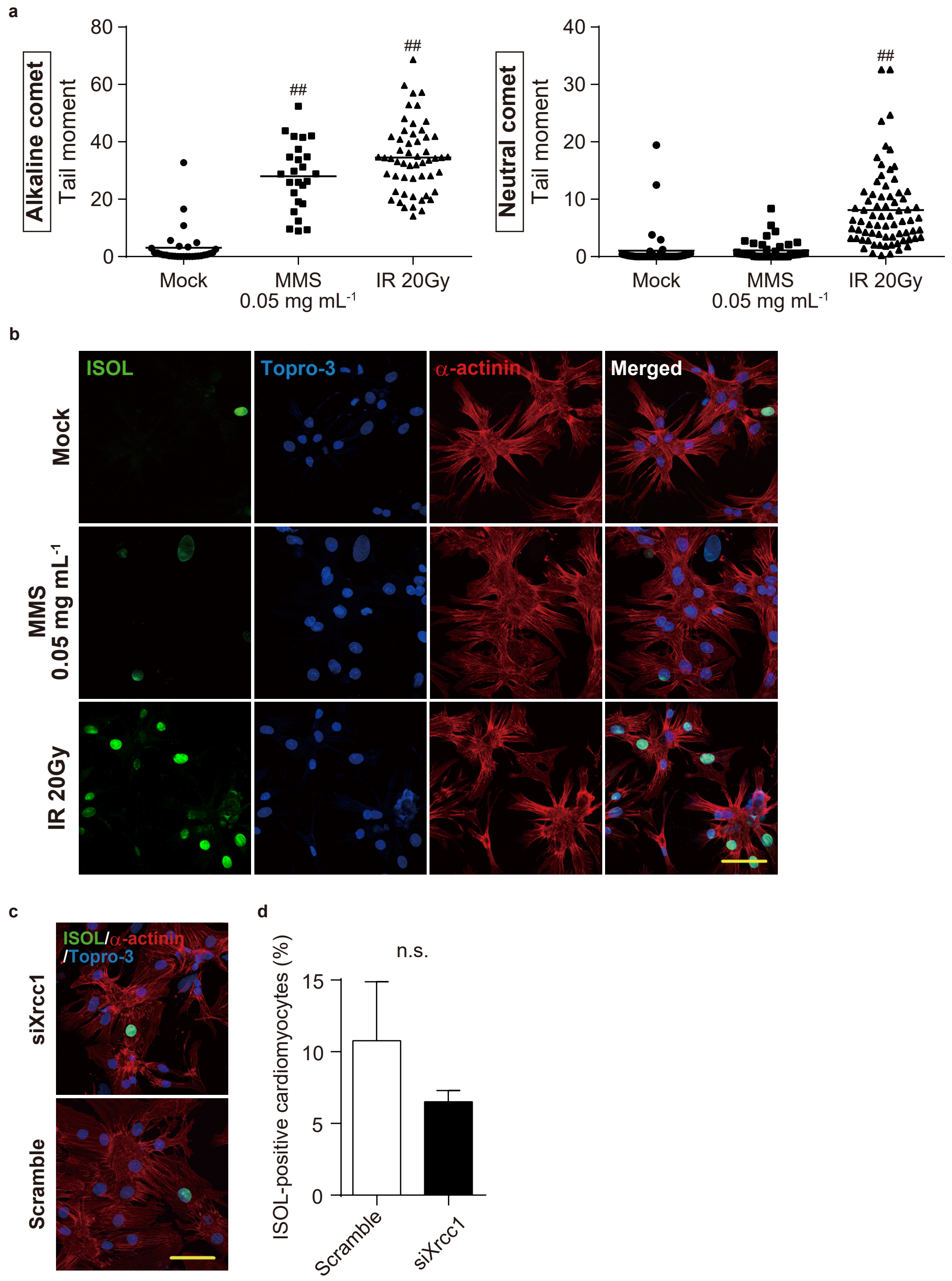

Supplementary Figure 6. Irradiation not MMS treatment or knockdown of Xrcc1 increases DNA DSB. a, b, Cultured neonatal rat cardiomyocytes (NRCMs) were treated with methyl methanesulfonate (MMS) (0.05 mg mL^{-1} for 10 min) to induce SSB[1] or irradiated (20 Gy) to induce DSB and the DNA damage was analyzed by comet assay (**a,** Alkaline comet: n=30, 25, 54; Neutral comet: n=44, 41, 72, respectively) and ISOL staining (**b**). Statistical significance was determined by Steel-Dwass test for **a**. ##p<0.01 versus Mock. ISOL-positive cells were stained green. Immunostaining for alpha-actinin (red) was used to label cardiomyocytes. Scale bar, 20 μm. **c, d,** NRCMs were transfected with siRNA against *Xrcc1* (siXrcc1) or scrambled negative control oligonucleotide (Scramble). ISOL staining (green) was performed 4 days after the knockdown of Xrcc1 (**c**) as described in **b.** The number of ISOL-positive cardiomyocytes was counted (**d,** n=5 each). Scale bar, 20 μm. Statistical significance was determined by Student's *t* test. Column and error bars show mean and s.e.m, respectively.

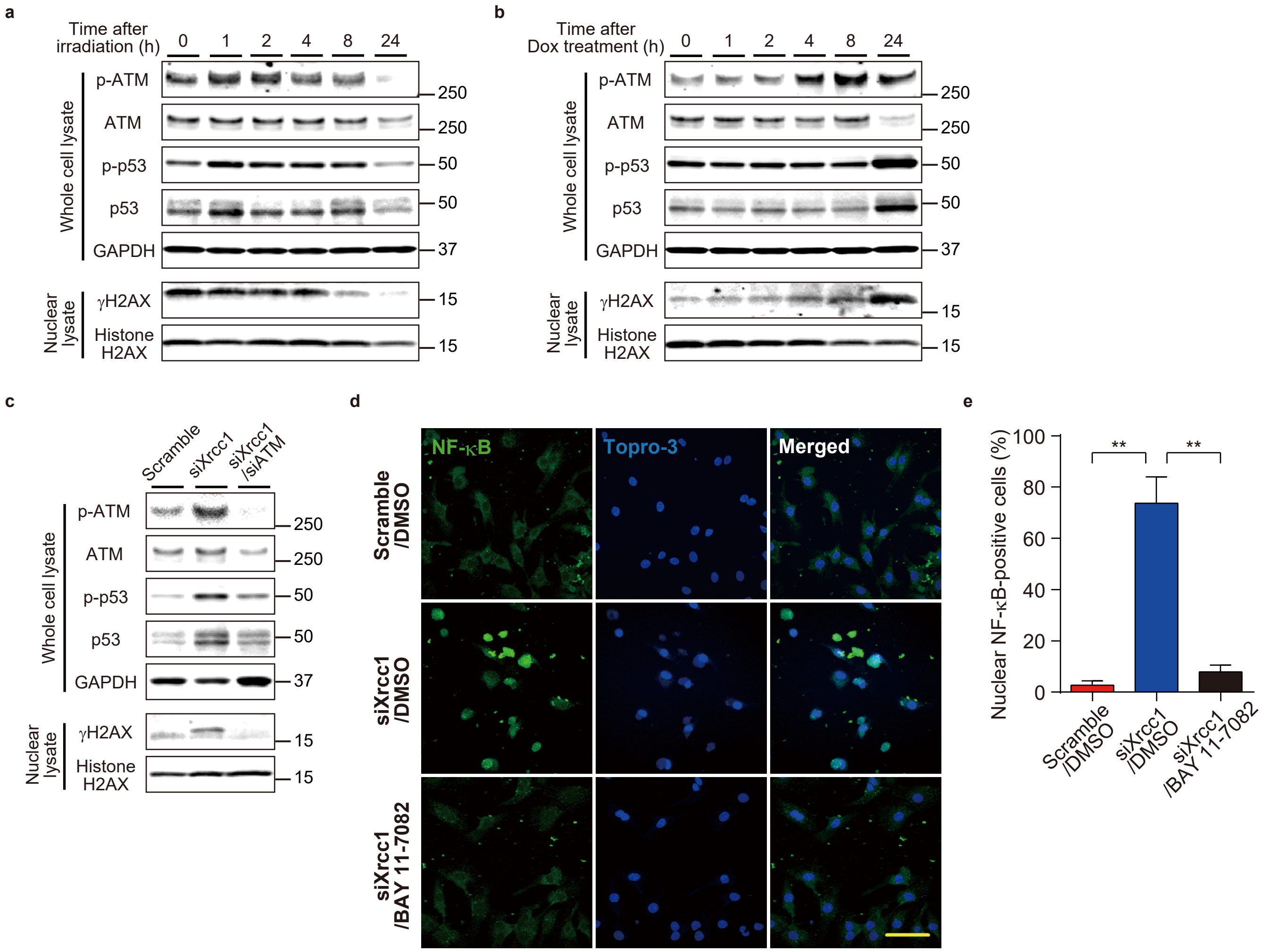

Supplementary Figure 7. Single-strand break increases the expression of inflammatory cytokine through activation of DDR and NF-κB. a, b, Neonatal rat cardiomyocytes (NRCMs) were irradiated (**a,** 20 Gy) or treated with doxorubicin (**b**, Dox, 1 μM for 3 h) and activation of DDR was assessed by western blotting against phospho- or total ATM, H2AX, and p53 at the indicated time points. Western blotting against GAPDH was used as a loading control. **c,** NRCMs were transfected with siRNA against *Xrcc1* (siXrcc1) alone or together with siRNA against *Atm* (siXrcc1/siATM). Four days after transfection, activation of DDR was assessed as described in **a, b**. **d, e,** NRCMs were transfected with siXrcc1 and treated with NF-κB inhibitor BAY 11-7082 (2 μM) or DMSO as a vehicle control. Nuclear translocation of NF-κB was assessed by immunofluorescence (**d,** green). The nuclei of the cells were counterstained with TO-PRO-3 iodide 642/661 (blue). Scale bar, 20 μm. Cells with positive nuclear NF-κB staining were counted (**e,** n=4). Statistical significance was determined by One-way ANOVA followed by the Tukey-Kramer HSD test. **p<0.01 between arbitrary two groups. Column and error bars show mean and s.e.m, respectively.

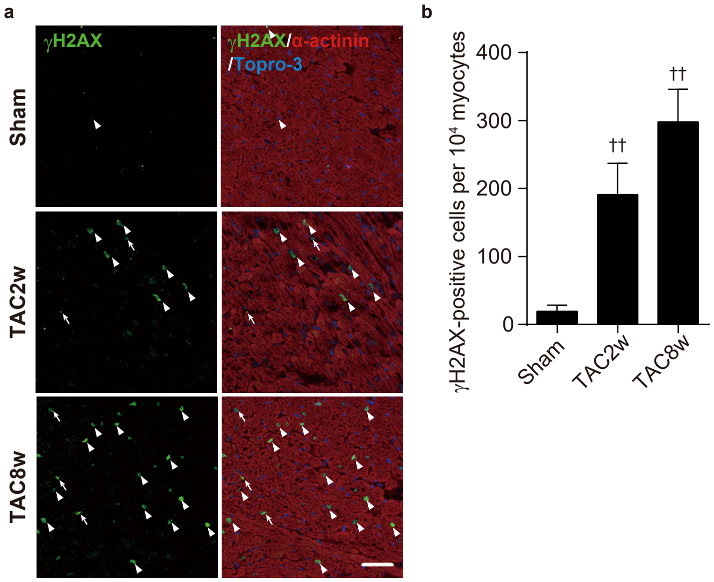

Supplementary Figure 8. Activation of persistent DDR in the failing heart. a, b, Activation of DDR after the TAC operation was assessed by immunostaining for phosphorylated H2AX (**a,** γH2AX, green). Immunostaining for alpha-actinin (red) was used to label cardiomyocytes. Arrowheads indicate γH2AX-positive cardiomyocytes and arrows indicate γH2AX-positive non-cardiomyocytes. Scale bar, 50 μm. The number of γH2AX-positive cardiomyocytes was counted at the indicated time points (**b,** n=6 each). Statistical significance was determined by One-way ANOVA followed by the Tukey-Kramer HSD test. ††p<0.01 versus Sham. Column and error bars show mean and s.e.m, respectively.

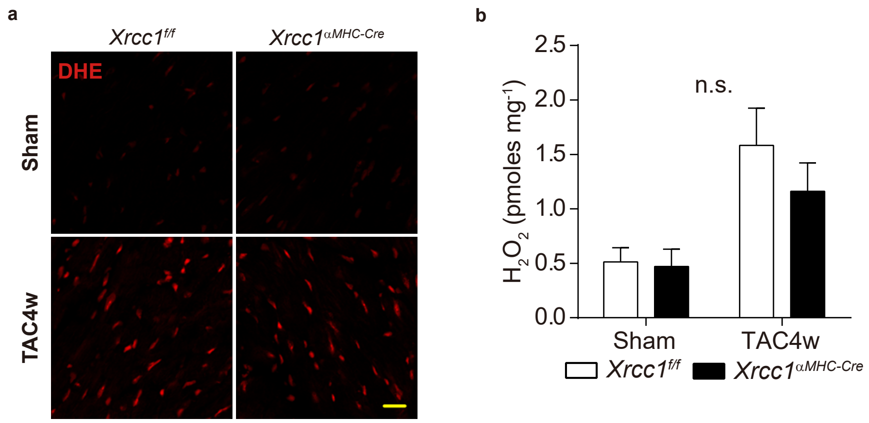

Supplementary Figure 9. Assessment of ROS production in the heart of cardiomyocyte-specific Xrcc1 deficient mice after pressure overload. **a,** Heart tissue sections of Sham- or TAC-operated *Xrcc1*$^{f/f}$ and *Xrcc1*$^{\alpha MHC\text{-}Cre}$ mice were incubated with dihydroethidium (DHE, 10 µM, red). Scale bar, 50 µm. **b,** The level of H_2O_2 in the heart of Sham- or TAC-operated *Xrcc1*$^{f/f}$ and *Xrcc1*$^{\alpha MHC\text{-}Cre}$ mice was measured using Amplex Red assay (n=4 each). Statistical significance was determined by One-way ANOVA followed by the Tukey-Kramer HSD test. Column and error bars show mean and s.e.m, respectively.

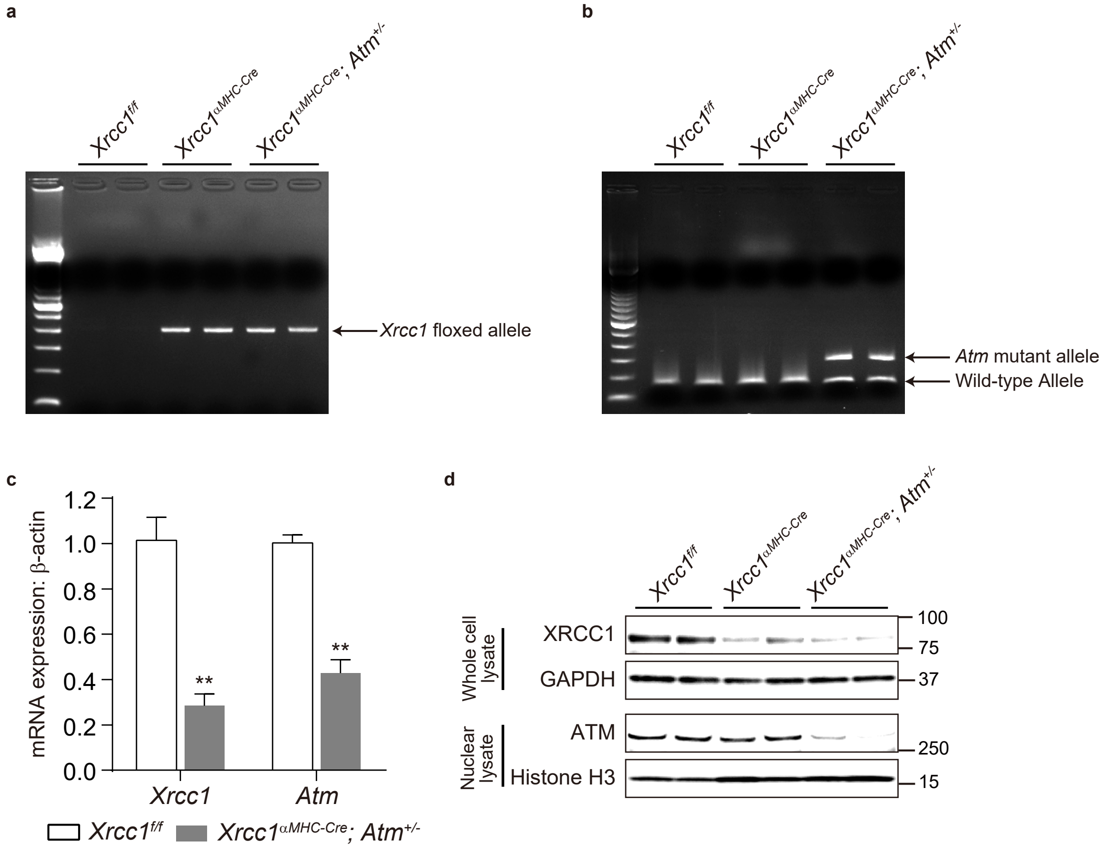

Supplementary Figure 10. Knockout confirmation in the heart of *Xrcc1$^{αMHC-Cre}$; Atm$^{+/-}$* mice. a-d, Genomic DNA, RNA and total protein was extracted from the heart tissue of *Xrcc1$^{f/f}$*, *Xrcc1$^{αMHC-Cre}$* and *Xrcc1$^{αMHC-Cre}$; Atm$^{+/-}$* mice. *Xrcc1* floxed alleles (**a**) and *Atm* mutant alleles (**b**) were detected by PCR, the expression levels of Xrcc1 and ATM were analyzed by real time PCR (**c,** n=4, 5 for each genotype, technical duplicates) and western blotting (**d**), respectively. Statistical significance was determined by Student's *t* test for **c**. **p<0.01 versus *Xrcc1$^{f/f}$* mice. Column and error bars show mean and s.e.m, respectively.

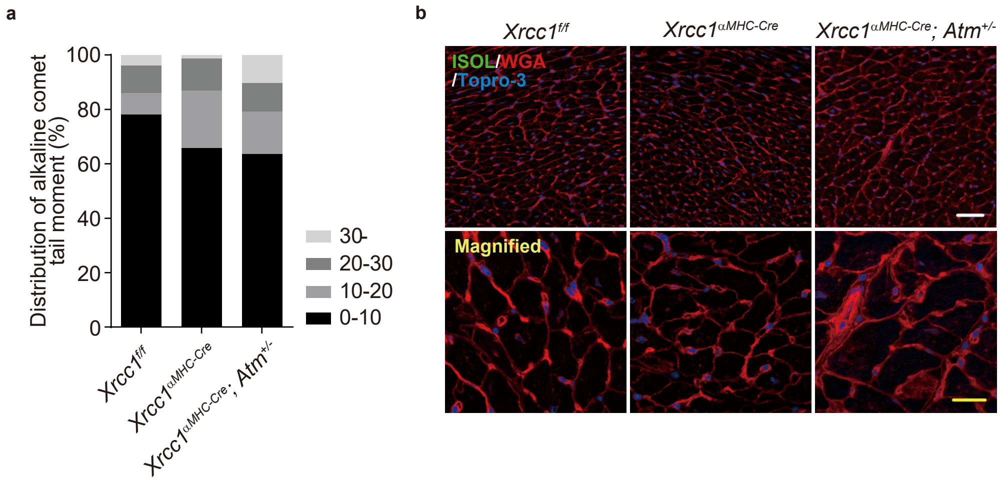

Supplementary Figure 11. Assessment of DNA SSB and DSB in the heart of *Xrcc1*$^{\alpha MHC\text{-}Cre}$ and *Xrcc1*$^{\alpha MHC\text{-}Cre}$; *Atm*$^{+/-}$ mice. a, Segmented bar charts of the alkaline comet tail moment described by dot plots in Fig. 6c. Cardiomyocytes with more comet tail moment tended to increase in *Xrcc1*$^{\alpha MHC\text{-}Cre}$ mice compared with *Xrcc1*$^{f/f}$ mice and remained in *Xrcc1*$^{\alpha MHC\text{-}Cre}$; *Atm*$^{+/-}$ mice (n=50, 76, 77, respectively). **b,** Fragmented DNA and DSB in cardiomyocyte**s** *Xrcc1*$^{f/f}$, *Xrcc1*$^{\alpha MHC\text{-}Cre}$ and *Xrcc1*$^{\alpha MHC\text{-}Cre}$; *Atm*$^{+/-}$ mice were labeled with ISOL staining (green). Wheat germ agglutinin (WGA, red) was used to visualize cardiomyocytes. White scale bar, 50 μm; yellow scale bar, 20 μm.

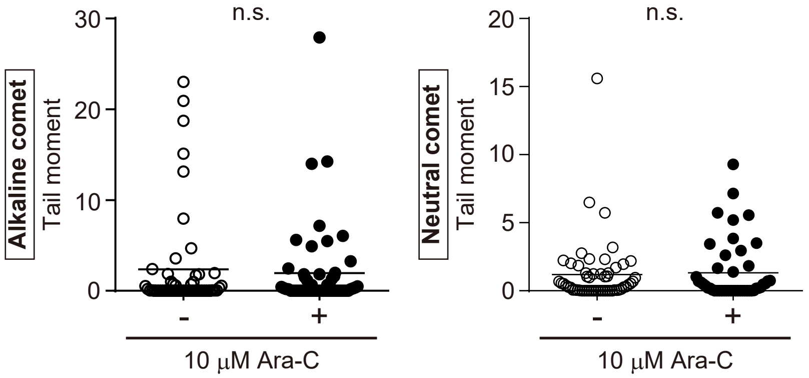

Supplementary Figure 12. Ara-C does not affect DNA strand break in cardiomyocytes. Neonatal rat cardiomyocytes were cultured with or without 10 μM cytosine β-D-arabinofuranoside hydrochloride (Ara-C). Four days later, the type and the level of DNA damage in cardiomyocytes was assessed by comet assay (Alkaline comet: n=52, 55; Neutral comet: n=55, 46, respectively). Statistical significance was determined by Mann-Whitney *U* test. Bars show mean.

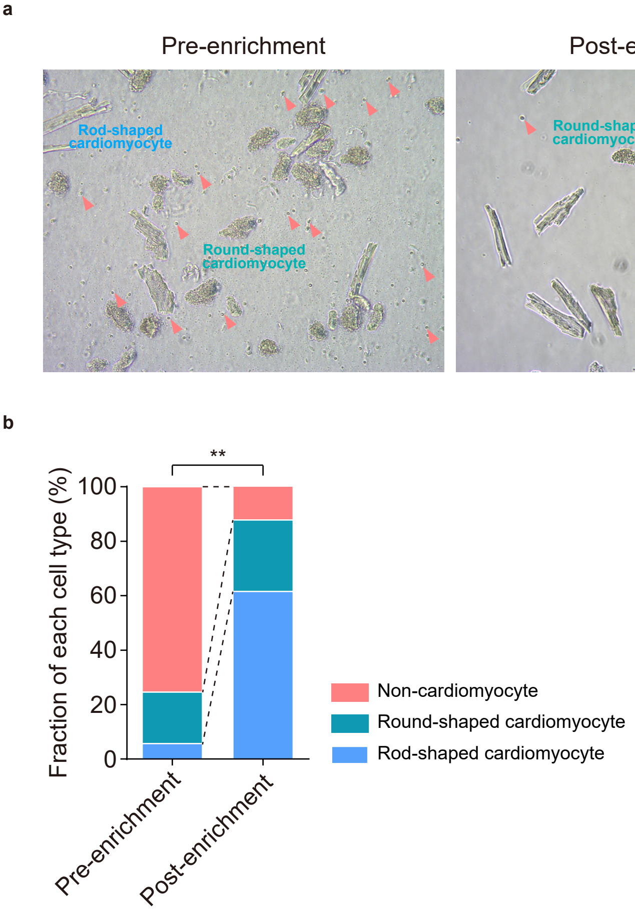

Supplementary Figure 13. Enrichment of cardiomyocytes using cell strainer. a, b, Both cardiomyocytes and non-cardiomyocytes were isolated using a Langendorff perfusion apparatus. Following isolation, cardiomyocytes were enriched by removing non-cardiomyocytes according to their size using cell strainer. Representative images of the cells before (Pre-enrichment) and after (Post-enrichment) enrichment (**a**). The number of cardiomyocytes and non-cardiomyocytes before and after enrichment was compared (**b,** n=9, 8 fields, respectively). Arrowheads indicate non-cardiomyocytes. Scale bar, 50 µm. Statistical significance was determined by Chi-squared test. **p<0.01 between pre- and post-enrichment.

Supplementary Figure 14

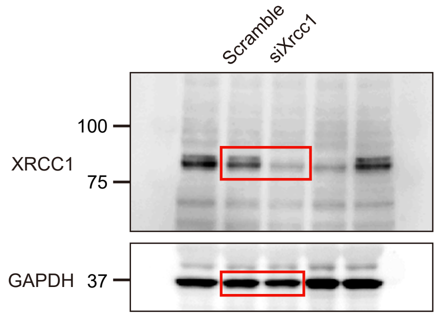

Uncropped membranes for Figure 3e

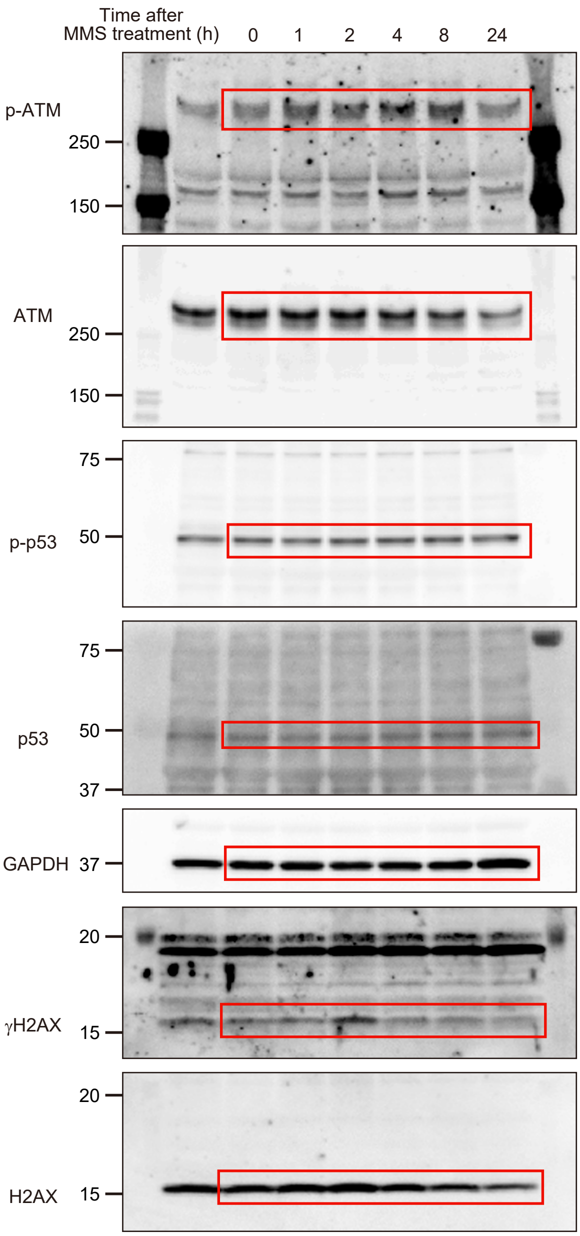

Uncropped membranes for Figure 4a

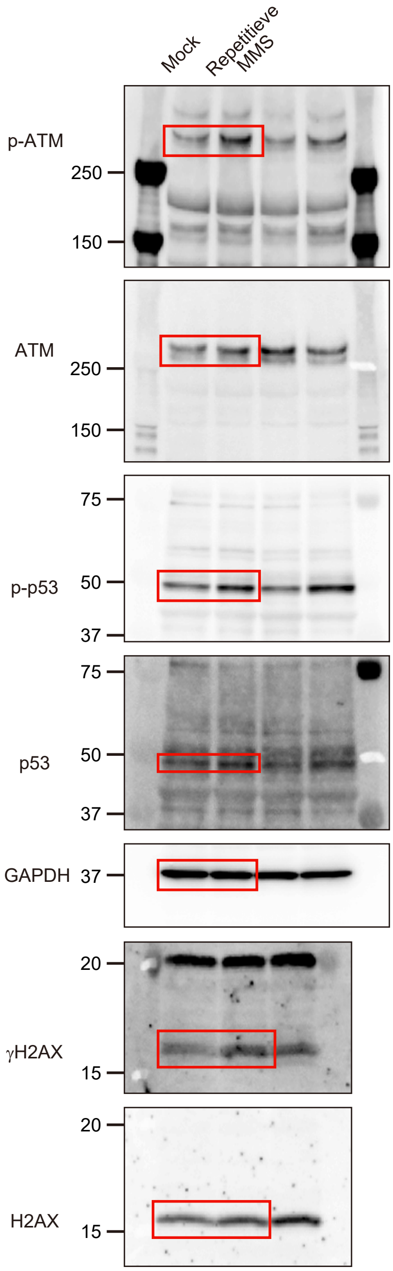

Uncropped membranes for
Figure 4b

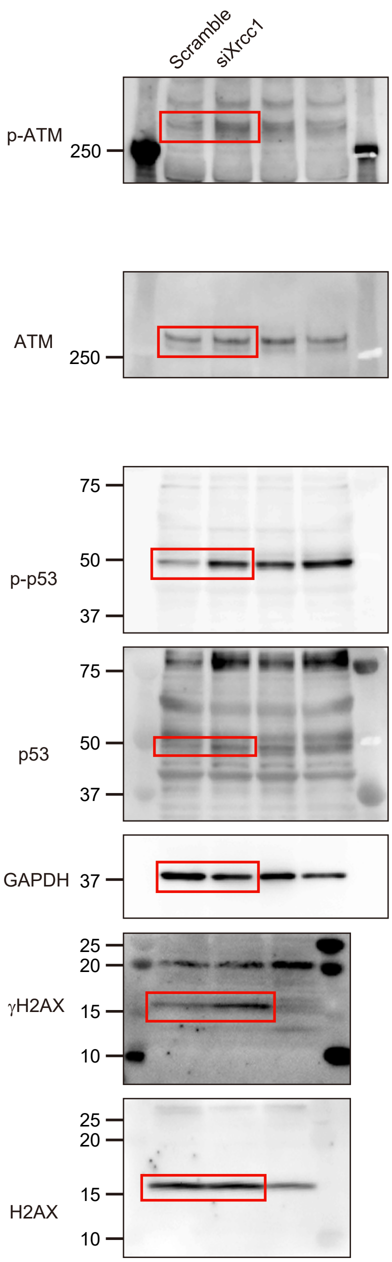

Uncropped membranes for
Figure 4c

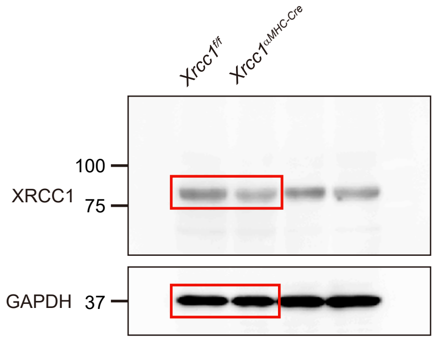

Uncropped membranes for Supplementary Figure 4c

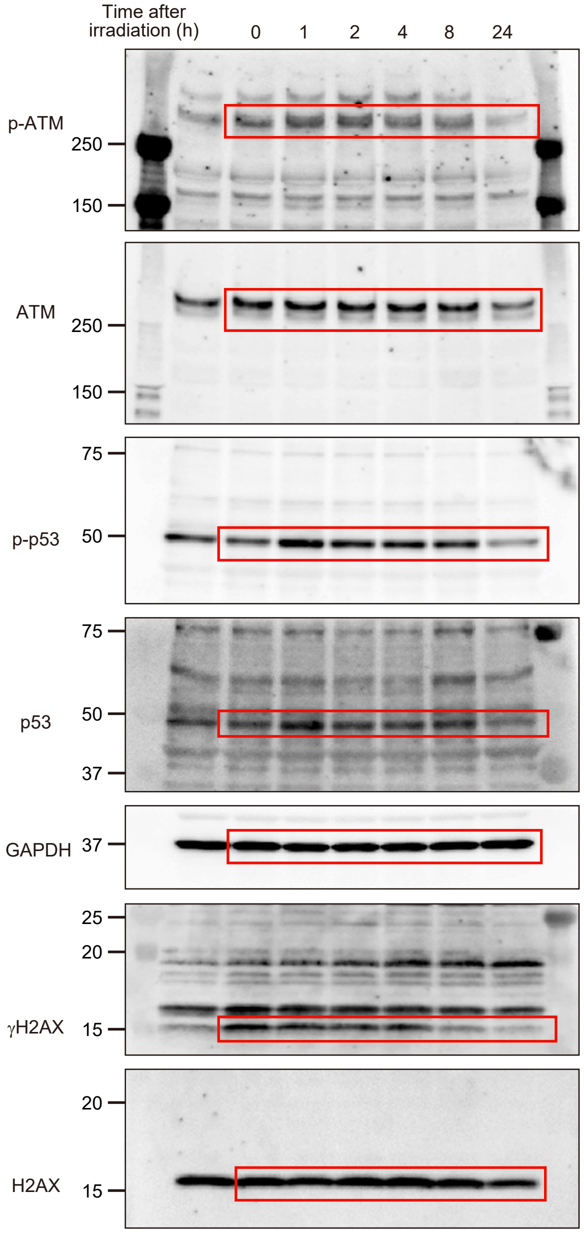

Uncropped membranes for
Supplementary Figure 7a

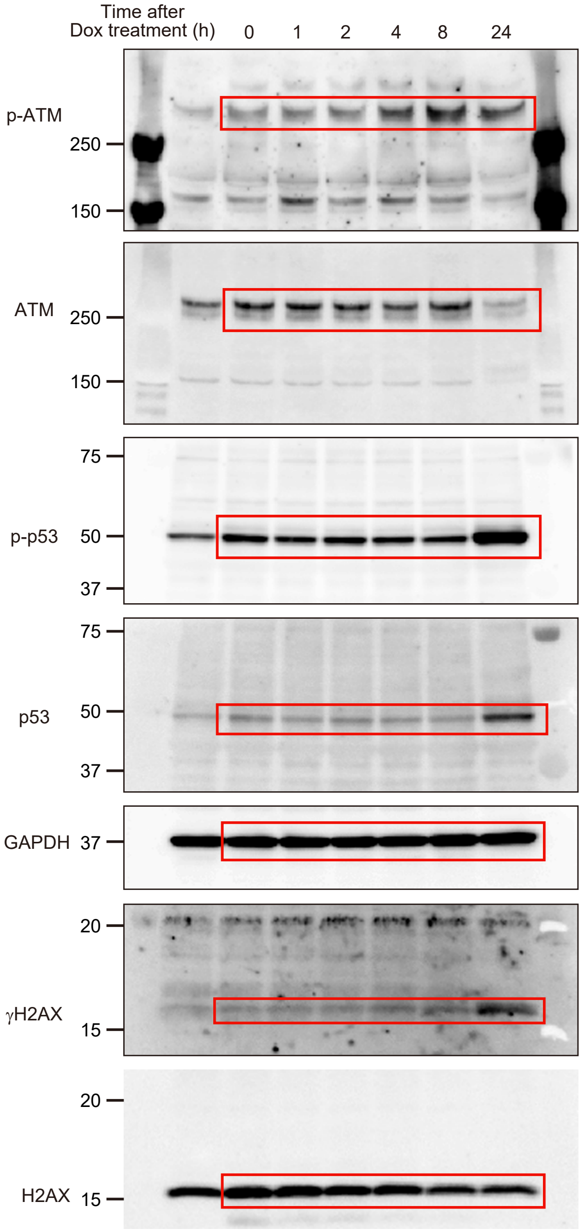

Uncropped membranes for
Supplementary Figure 7a

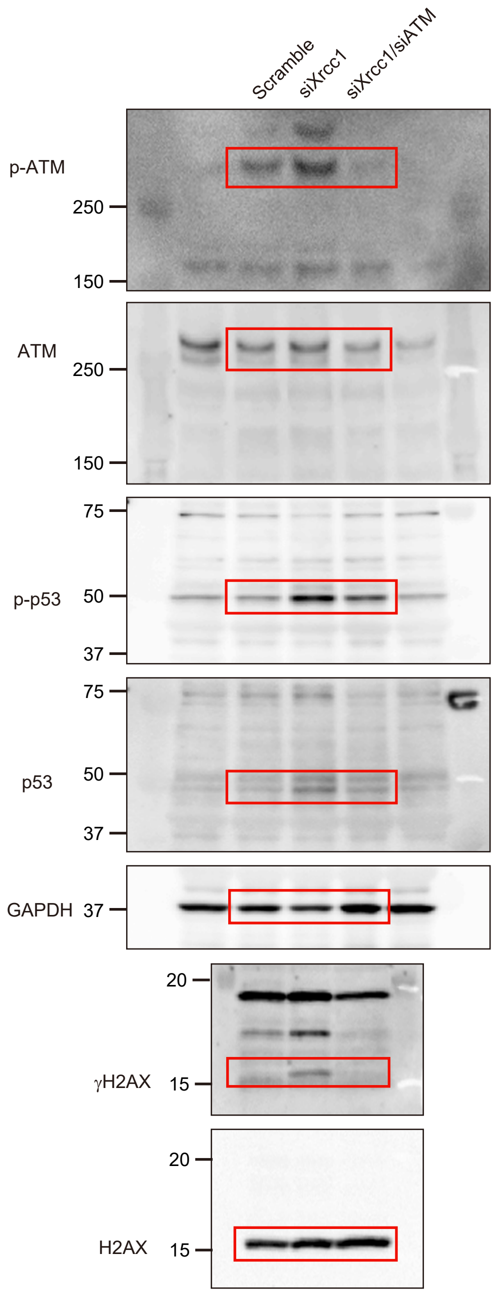

Uncropped membranes for Supplementary Figure 7c

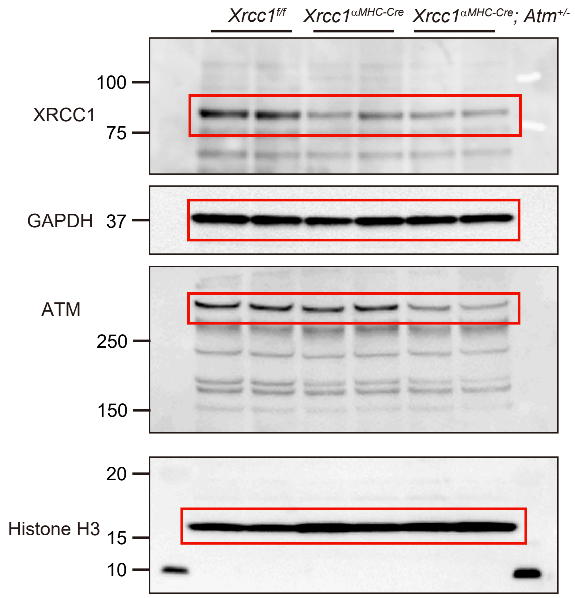

Uncropped membranes for Supplementary Figure 10c

Supplementary References

1 Pascucci, B., Russo, M. T., Crescenzi, M., Bignami, M. & Dogliotti, E. The accumulation of MMS-induced single strand breaks in G1 phase is recombinogenic in DNA polymerase beta defective mammalian cells. *Nucleic acids research* **33**, 280-288, doi:10.1093/nar/gki168 (2005).
